# The IMproving Preclinical Assessment of Cardioprotective Therapies (IMPACT): multicenter pig study on the effect of ischemic preconditioning

**DOI:** 10.1007/s00395-024-01083-9

**Published:** 2024-10-18

**Authors:** Petra Kleinbongard, Carlos Galán Arriola, Lina Badimon, Veronica Crisostomo, Zoltán Giricz, Mariann Gyöngyösi, Gerd Heusch, Borja Ibanez, Attila Kiss, Dominique P. V. de Kleijn, Bruno K. Podesser, Rafael Ramírez Carracedo, Antonio Rodríguez-Sinovas, Marisol Ruiz-Meana, Francisco M. Sanchez Margallo, Gemma Vilahur, José Luis Zamorano, Carlos Zaragoza, Peter Ferdinandy, Derek J. Hausenloy

**Affiliations:** 1 Institute for Pathophysiology, West German Heart and Vascular Center, University of Essen Medical School, University of Duisburg-Essen, Hufelandstr. 55, 45122 Essen, Germany; 2 Centro Nacional de Investigaciones Cardiovasculares Carlos III, CIBER de Enfermedades Cardiovasculares (CIBERCV), Melchor Fernández Almagro 9, 28029 Madrid, Spain; 3 Research Institute Hospital de La Santa Creu I Sant Pau-IIB Sant Pau, and CIBER Enfermedades Cardiovasculares, Barcelona, Spain; 4 Cardiovascular Area, Jesús Usón Minimally Invasive Surgery Centre (CCMIJU), Cáceres, Spain; 5 CIBER de Enfermedades Cardiovasculares (CIBERCV), RICORS-TERAV Network, ISCIII, Madrid, Spain; 6 Cardiovascular and Metabolic Research Group, Department of Pharmacology and Pharmacotherapy, Semmelweis University, Budapest, Hungary; 7 Pharmahungary Group, Szeged, Hungary; 8 Division of Cardiology, Department of Internal Medicine II, Medical University of Vienna, 1090 Vienna, Austria; 9 Centro Nacional de Investigaciones Cardiovasculares (CNIC), Madrid, Spain; 10 Ludwig Boltzmann Institute for Cardiovascular Research at the Center for Biomedical Research and Translational Surgery, Medical University of Vienna, Vienna, Austria; 11 Department of Vascular Surgery, University Medical Center Utrecht, Utrecht, The Netherlands; 12 Unidad de Investigación Cardiovascular, Departamento de Cardiología, Hospital Ramón y Cajal (IRYCIS), Universidad Francisco de Vitoria, Madrid, Spain; 13 Cardiovascular Diseases Research Group, Department of Cardiology, Vall d’Hebron Institut de Recerca (VHIR), Hospital Universitari Vall d’Hebron, Barcelona, Spain; 14 Centro de Investigación Biomédica en Red de Enfermedades Cardiovasculares (CIBERCV), Instituto de Salud Carlos III (ISCIII), Madrid, Spain; 15 Jesús Usón Minimally Invasive Surgery Centre (CCMIJU), Cáceres, Spain; 16 University Hospital Ramon Y Cajal, Madrid, Spain; 17 Department of Pharmacology and Pharmacotherapy, Semmelweis University, Nagyvárad Tér 4, Budapest, 1089 Hungary; 18 Cardiovascular and Metabolic Disorders Program, Duke-NUS Medical School, 8 College Road, Singapore, 169857 Singapore; 19 National Heart Research Institute Singapore, National Heart Centre, Singapore, Singapore; 20 Yong Loo Lin School of Medicine, National University Singapore, Singapore, Singapore; 21 The Hatter Cardiovascular Institute, University College London, London, UK; 22 Center for Pharmacology and Drug Research and Development, Semmelweis University, Budapest, Hungary

**Keywords:** Acute myocardial infarction, Ischemia/reperfusion injury, Ischemic preconditioning, Multicenter network, Pig, Randomized-controlled trial

## Abstract

Numerous cardioprotective interventions have been reported to reduce myocardial infarct size (IS) in pre-clinical studies. However, their translation for the benefit of patients with acute myocardial infarction (AMI) has been largely disappointing. One reason for the lack of translation is the lack of rigor and reproducibility in pre-clinical studies. To address this, we have established the European IMproving Preclinical Assessment of Cardioprotective Therapies (IMPACT) pig AMI network with centralized randomization and blinded core laboratory IS analysis and validated the network with ischemic preconditioning (IPC) as a positive control. Ten sites in the COST Innovators Grant (IG16225) network participated in the IMPACT network. Three sites were excluded from the final analysis through quality control of infarct images and use of pre-defined exclusion criteria. Using a centrally generated randomization list, pigs were allocated to myocardial ischemia/reperfusion (I/R, *N* = 5/site) or IPC + I/R (*N* = 5/site). The primary endpoint was IS [% area-at-risk (AAR)], as quantified by triphenyl-tetrazolium-chloride (TTC) staining in a centralized, blinded core laboratory (5 sites), or IS [% left-ventricular mass (LV)], as quantified by a centralized, blinded cardiac magnetic resonance (CMR) core laboratory (2 sites). In pooled analyses, IPC significantly reduced IS when compared to I/R (57 ± 14 versus 32 ± 19 [%AAR] *N* = 25 pigs/group; *p* < 0.001; 25 ± 13 versus 14 ± 8 [%LV]; *N* = 10 pigs/group; *p* = 0.021). In site-specific analyses, in 4 of the 5 sites, IS was significantly reduced by IPC when compared to I/R when quantified by TTC and in 1 of 2 sites when quantified by CMR. A pig AMI multicenter European network with centralized randomization and core blinded IS analysis was established and validated with the aim to improve the reproducibility of cardioprotective interventions in pre-clinical studies and the translation of cardioprotection for patient benefit.

**Supplementary Information:**

The online version contains supplementary material available at 10.1007/s00395-024-01083-9.

## Introduction

Despite advances in the management and treatment of acute myocardial infarction (AMI) by reperfusion, the 1-year mortality is still high. The 1-year mortality rate is below < 10% in clinical trials [19, 42, 47, 55]. However, in large European registries, which may more accurately reflect real-world conditions, the mortality rate varies and may range up to 15–21%. [4, 31, 57]. Thus, there is still a need for new cardioprotective interventions beyond timely reperfusion to reduce myocardial infarct size (IS), prevent the development of heart failure, and improve clinical outcomes [26, 29]. Intensive investigation and elucidation of the mechanisms underlying acute myocardial ischemia/reperfusion (I/R) injury have resulted in the identification of a large number of cardioprotective targets and interventions [2, 9, 10, 16, 17, 21, 24, 27, 40]. Although many of these treatments have been demonstrated to reduce IS in pre-clinical animal studies [24], their translation into the clinical setting for patient benefit has been largely disappointing [18, 24, 25, 30].

Obvious differences exist between the pre-clinical animal studies, clinical trials, and clinical practice [22, 25, 31], and several reasons have been proposed to explain the translational failure of cardioprotection into the clinic. These include age and comorbidities (such as diabetes, hypertension, and hyperlipidemia) and concomitant medications (such as anti-platelet agents and statins) which are present in AMI patients and may interfere with cardioprotective signaling and efficacy [7, 13, 37]. In many pre-clinical studies, these have not been taken into account in the study design. Similarly, the lack of rigor and reproducibility in the design and conduct of the pre-clinical animal studies may play a role [5, 6, 22, 45, 53].

To address the latter, the EU-CARDIOPROTECTION COST Action (CA16225) network has published guidelines to improve the rigor and robustness of pre-clinical cardioprotection studies [8] and proposed the IMproving Preclinical Assessment of Cardioprotective Therapies (IMPACT) criteria and step-by-step framework [45]. The use of pigs has been proposed as the most appropriate species in this translation pathway because of their high anatomical and physiological similarity to humans, i.e., comparable hemodynamics and spatial and temporal evolution of recent AMI [8, 32]. As in AMI patients, IS should be used as the most relevant endpoint for assessing cardioprotective efficacy. IS should be quantified by histochemistry as a fraction of the area-at-risk (AAR) via triphenyl-tetrazolium-chloride (TTC) staining and AAR demarcation with sodium fluorescein or a blue dye—the gold-standard setup for pre-clinical models [8] and/or by cardiac magnetic resonance imaging (CMR), the latter reflecting the gold-standard approach for assessing IS in AMI patients [8, 28, 34]. A key aspect of the IMPACT criteria is to establish a network of research centers capable of conducting multicenter pig AMI studies in a centralized, randomized, blinded manner, similar to the design of randomized-controlled clinical trials [45].

The realization of how important such a network is for improving translation and the idea of setting up such a network is not novel. Two similar networks have been initiated. The National Heart, Lung, and Blood Institute (NHLBI)-funded, Consortium for preclinicAl assESsment of cARdioprotective interventions (CAESAR) research network of 3 sites performed AMI in mice, rabbits, and pigs and evaluated previously established pharmacological cardioprotective strategies (i.e., sildenafil, sodium nitrite, and chloramphenicol succinate). The network failed to demonstrate cardioprotection with these pharmacological treatments but did manage to show a reduction in IS with ischemic preconditioning (IPC) [5, 35, 44, 46]. Due to lack of funding, the CAESAR consortium is no longer functioning, but it did succeed in demonstrating the utility of a multicenter network for evaluating the reproducibility of novel cardioprotective interventions. More recently, the Spanish CIBER-CLAP (CIBERCV Cardioprotection Large Animal Platform) was set up to undertake pig AMI multicenter studies for evaluating cardioprotective therapies [50], but no results have yet been published.

We here aimed to establish a European pig AMI multicenter network with centralized randomization and blinded core laboratory analysis of IS by TTC and CMR. To validate this network, we used IPC as the cardioprotective stimulus, which has been established as the strongest and most robust stimulus for cardioprotection [24]. To increase the translational value of our multicenter study, pigs of different breeds and with different housing conditions, male and female as well as juvenile and adult pigs were included. The experimental protocol, e.g., anesthesia and/or the duration of ischemia, was also not standardized and the different sites used the protocol established at their sites. With this approach, we aimed to reproduce the heterogeneous conditions in the clinical setting. After initiation of the network, but prior to the start of the studies, we subjected each site to rigorous quality control (QC) of the TTC and CMR infarct images by core laboratories (in Germany and Spain) within a predetermined time frame.

## Materials and methods

### Study design

The IMPACT pig AMI multicenter network was centrally coordinated by a working group comprising: (1) the principal investigator and team members from each of the participating sites; (2) the IMPACT centralized core laboratories for IS analysis by triphenyl-tetrazolium-chloride (TTC; University of Duisburg-Essen, Essen, Germany) and CMR (CNIC, Madrid Spain); and (3) the IMPACT central statistical core (Duke-NUS/NHCS, Singapore) who provided the central randomization lists to the sites and undertook all statistical analyses.

The experimental protocols conformed to the EU directive 2010/63EU on the protection of animals used for scientific purposes and the Animal Research: Reporting of In Vivo Experiments (ARRIVE) guidelines [49]. The experimental protocols were formally approved by the appropriate national or institutional ethics committees.

**Fig. 1:**
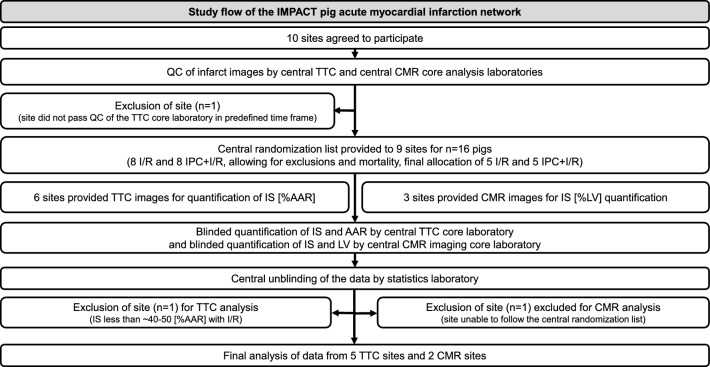
Study flow of IMPACT pig acute myocardial infarction multicenter network. Ten sites agreed to participate in the IMproving Preclinical Assessment of Cardioprotective Therapies (IMPACT) pig acute myocardial infarction network. One site was excluded as it did not pass the QC. Central randomization lists were provided to 9 sites to undertake I/R and IPC + I/R studies. Six sites used TTC images for central blinded core laboratory quantification of IS [%AAR] and 3 sites used CMR for central blinded core laboratory quantification of infarct size [% LV]. One TTC site was excluded as the AAR was smaller than the pre-defined criterion and one CMR site was excluded as it did not follow the central randomization list. Five TTC sites and 2 CMR sites underwent final analysis of infarct size data. *AAR* area-at-risk, *CMR* cardiac magnetic resonance imaging, *IPC* ischemic preconditioning, *I/R* ischemia/reperfusion, *IS* infarct size, *LV* left ventricle, *QC* quality control

Ten participants of the COST Innovators Grant (IG16225) network agreed to participate in establishing the IMPACT pig AMI network: 5 from Spain, 2 from Austria, 1 from Germany, 1 from the Netherlands, and 1 from Hungary (Table 1). All participating sites had previously established a pig AMI model in their laboratories (for references, see Data sheet experimental design). Prior to the onset of the studies for the IMPACT network, each site underwent rigorous QC assessment of their TTC and CMR infarct images by the core laboratories in a predetermined time frame, with individual feedback provided to each site for optimization of the infarct images (see QC checklist in the Suppl. Table 1, and the overall process in Fig. 1). One site did not pass the QC within the predetermined time frame and was excluded from the actual study (Fig. 1). After passing QC, the IMPACT central statistical core generated central randomization lists for each site to assign pigs to I/R or IPC + I/R. Finally, *N* = 5 I/R and *N* = 5 IPC + I/R experiments per site were performed (Fig. 1). The selection of 5/5 animals per site was based on a recent study of one of the participating sites, which was based on a power analysis [38]. If an animal died during the experiment or was excluded due to the pre-defined criteria, further experiments were conducted to reach a total of 5 pigs per site. Only one exclusion criterion applied to all sites; conspicuous, unhealthy pigs were not included. For all other site-specific exclusion criteria, please see the Suppl. Data sheet experimental design.

**Table 1:** Summary table with the participating sites and information on pigs, pre-treatment protocols, surgical preparation, IPC protocol, ischemia and reperfusion times, and infarct size quantification method

Participating sites	Pigs			(Pre-)treatment			Preparation	IPC	Ischemia	Reperfusion	Infarct size and area-at-risk
				Anesthetics/analgesic regime		Antiarrhythmics		3 cycles		hours (h)/days	
Vall d´Hebron, Barcelona, Spain	Farm pig	Juvenile	Male	Fentanyl	Sodium thiopental	No	Open chest	5/10 min	45 min	3 h	TTC/fluorescein
Essen, Germany	Mini pig	Adult	Female/male	Isoflurane	Sufentanil	No	Open chest	5/10 min	60 min	3 h	TTC/blue dye
CBR, Vienna, Austria	Farm pig	Juvenile	Male	Sevoflurane	Fentanyl	Yes	Closed chest	5/5 min	60 min	3 h	TTC/blue dye
Utrecht, The Netherlands	Farm pig	Juvenile	Female	Midazolam	Sufentanil	Yes	Closed chest	5/5 min	60 min	3 days	TTC/blue dye
IIB-Sant Pau, Barcelona, Spain	Farm pig	Juvenile	Female	Isoflurane	Buprenorphine/fentanyl	Yes	Closed chest	5/5 min	90 min	5 days	TTC/blue dye
CCMIJU, Cáceres, Spain	Farm pig	Juvenile	Female	Sevoflurane	Ketorolac	Yes	Closed chest	5/5 min	90 min	5–7 days	TTC/blue dye
UFV, Madrid, Spain	Farm pig	Juvenile	Female	Isoflurane/propofol	Fentanyl/diazepam	Yes	Closed chest	5/5 min	45 min	7 days	TTC/blue dye
Dep Med II, Vienna, Austria	Farm pig	Juvenile	Female/male	Isoflurane	–	Yes	Closed chest	5/5 min	90 min	4–7 days	CMR
CNIC, Madrid, Spain	Farm pig	Juvenile	Female	Ketamine/xylazine/midazolam	Buprenorphine/bupivacain	Yes	Closed chest	5/5 min	45 min	6 days	CMR
Budapest, Hungary	Farm pig	Juvenile	Female	Isoflurane	Burprenorphine	Yes	Closed chest	5/5 min	90 min	7 days	CMR

For details, please see the “Suppl. Data sheet experimental design”

*CBR* Center for Basic Research, *CCMIJU* Centro de Cirugía de Mínima Invasión Jesús Usón, *CNIC* Centro Nacional de Investigaciones Cardiovasculares Carlos III, *CMR* cardiac magnetic resonance imaging, *Dep Med II* Department of Medicine II, *IIB-Sant Pau* Sant Pau Biomedical Research Institute, *IPC* ischemic preconditioning, *TTC* triphenyl-tetrazolium-chloride, *UFV* Universidad Francisco de Vitoria

### Study procedures

All participating sites used procedures which were established in their laboratories for cardioprotection research in the pig AMI model. Thus, there was limited standardization of experimental conditions and study protocols. Site-specific details of these parameters are provided in Table 1 and the Suppl. Data sheet experimental design. All sites used juvenile farm pigs (different breeds), except for one site which used adult minipigs. Five sites included only female pigs, 2 sites only male pigs, and 2 sites both female and male pigs (Table 1). Pigs were anesthetized with inhalational anesthesia (6 sites) or intravenous anesthesia (3 sites, Table 1). Barbiturates, opioids, or non-opioid analgesics were used for analgesia (Table 1). Two sites performed an open-chest AMI preparation, one site used colored microspheres to quantify post-mortem the regional myocardial blood flow [43], and the other site measured coronary flow online via a Doppler coronary flow probe [1]. A high blood flow during ischemia and a low blood flow during reperfusion were used as a-priori inclusion criteria; for details, see Suppl. Data sheet experimental design. Eight sites used a closed-chest AMI preparation and only these sites used anti-arrhythmic drugs (Table 1). The left anterior descending (LAD) coronary artery was the standard occlusion site. For TTC measurement, the duration of coronary occlusion targeted for an IS of 40–50 [%AAR], and sites with an IS substantially less than 40% of AAR were excluded from further analysis.

The duration of ischemia ranged between 45 and 90 min. Three cycles of I/R prior to the index ischemia were standardized as the IPC maneuver. Two sites used 5/10 min cycles and the other 8 sites used 5/5 min cycles (Table 1). The duration of reperfusion ranged from 3 h up to 7 days (Table 1). Fibrillation episodes and defibrillation shocks used during IPC + I/R and I/R protocols are listed in Suppl. Table 2. TTC protocols and the CMR acquisition protocols were not standardized across sites (Suppl. Data sheet experimental design).

### Study endpoints

The primary endpoint was myocardial IS as fraction of the AAR [%AAR] as measured via TTC or as fraction of the left-ventricular (LV) mass as measured via CMR [%LV]. IS was analyzed by the TTC core laboratory (University of Duisburg-Essen, Essen, Germany) or by the CMR core laboratory (CNIC, Madrid, Spain).

The TTC staining protocol was not standardized, and the sites used their established protocols (for details, please see the Suppl. Data sheet experimental design). Although TTC staining is considered to be the gold-standard setup for pre-clinical models [8], staining with TTC sometimes gives ambiguous results, i.e., areas that are neither white/yellow nor bright red, but “pink”. Here, we quantified all areas that were not clearly red, i.e., vital areas, as infarct (for examples see Suppl. Figure 1). TTC staining was used to quantify IS not only after short reperfusion times (hours) but also after longer reperfusion times (days); for details, please see the Suppl. Data sheet experimental design. TTC staining has only been validated for reperfusion times of minutes/hours [15], and we did not validate the quality of TTC staining further by histologic staining in our present study. TTC images were quantified using digital planimetry (ImageJ 1.54d; National Institutes of Health). Using the scale present in each original image, the following areas were calculated and averaged for both sides of each slice: total area of the LV, the AAR (stained with sodium fluorescein or a blue dye), and the area of TTC-negative tissue (infarcted). The AAR was calculated as fraction of the LV [%LV], and the IS was calculated as a fraction of the AAR [%AAR] and the LV [%LV], respectively [8]. All TTC analyses were performed by investigators who were blinded to the treatment allocation.

Baseline CMR scans were performed 0–4 days before coronary occlusion, and scans were repeated at 6–7 days post-infarction before sacrifice (Suppl. Data sheet experimental design). The end-diastolic and end-systolic phases of the cardiac cycle were defined as those in the frames of maximum and minimum LV diameter, respectively. For intracardiac LV volume measurements, the endocardial border of the sub-valvular zone to the apex was manually traced, excluding trabeculae and papillary muscles, which remained within the blood pool. For the determination of cardiac LV mass [ml volume], the epicardial border was traced in all slices during the end-diastolic phase, which is more reliable for accurate determination. Left-ventricular ejection fraction (LVEF) was calculated as the difference between end-systolic and end-diastolic volumes as fraction of end-diastolic volume [%]. For IS determination [%LV], the endo- and epicardial borders were delineated in a mid-diastolic phase at the late gadolinium enhancement (LGE) sequence. By placing a region of interest in the non-infarcted remote area, the scar was semi-automatically established as anything deviating by 6 or more standard deviations from the histogram. Extension of edema [%LV] was similarly measured to LGE but in the T2-weighted sequence, using a threshold of 6 standard deviations in intensity compared to the remote area. Since the conventional CMR sequences traditionally used for measuring the AAR in the experimental setting may be affected by post-infarct stages and the use of cardioprotective therapies [14], the AAR was determined using contrast computed tomography [34] for the CNIC, Madrid, Spain group and the Bypass Angioplasty Revascularization Investigation Myocardial Jeopardy (BARI) score from angiography [48] for the Budapest, Hungary group. CMR images were analyzed by dedicated IntelliSpace Portal software (Philips, the Netherlands) to ensure QC of all sequences. For the enhanced sequences, analysis was conducted semi-automatically. All CMR analyses were performed by investigators who were blinded to the treatment allocation.

### Central randomization and statistical analyses

The IMPACT central statistics laboratory provided the central randomization list to each site, performed data unblinding following completion of all experiments, and undertook the statistical analysis for all data. The sequence of treatments (I/R or IPC + I/R) was randomly permuted, and a list was generated for each site to use up to 16 animals (*N* = 8 I/R, *N* = 8 IPC + I/R), thus allowing for exclusions and mortality. Myocardial AAR [%LV], IS [%AAR], and IS [%LV] were analyzed separately. Data were tested for normal distribution using the Shapiro–Wilk test. AAR and IS were analyzed using the Wilcoxon rank-sum test, and for the pooled data analysis, a two-way ANOVA in STATA (StataCorp. 2023. Stata Statistical Software: Release 18. College Station, StataCorp LLC, Texas, USA) was used. Statistical significance was set at *p* < 0.05.

The intraclass correlation coefficient (ICC) among sites was calculated with R (software version 4.3.2; The R Foundation for Statistical Computing, Vienna, Austria) using the ‘icc’ package (Gamer et al., 2012. icc: Various Coefficients of Interrater Reliability and Agreement. R package version 0.84.1). Effect sizes and type II errors on IS data from each site and the pooled IS data were calculated using G-Power 3.1.9.7 (University of Düsseldorf, Germany, 2020).

## Results

### Study exclusions and mortality during the experimental procedure

Details of animal exclusions based on pre-defined criteria and the death of animals during the experimental procedure are listed in Suppl. Table 3. One site was excluded from TTC analysis (Fig. 1), as IS was substantially less than 40 [%AAR], the mean value was 17 ± 14 [%AAR] (Suppl. Figure 2B). Another site was excluded from CMR analysis, because the site did not follow the central randomization list due to logistical reasons (Fig. 1).

### Cardioprotective efficacy of IPC assessed by TTC

**Fig. 2:**
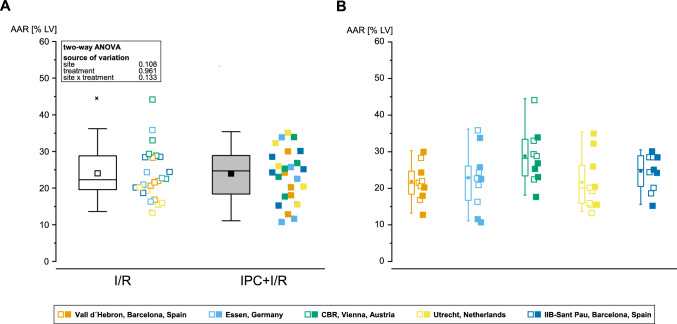
Pooled and site-specific analysis of area-at-risk from the 5 sites providing triphenyl-tetrazolium-chloride images. **A** Pooled analysis of AAR quantified by histochemistry (fluorescein or blue dye) was comparable between I/R (open squares) and IPC + I/R (filled squares). **B** Site-specific analysis of AAR was comparable between the 5 sites. Data are presented as minimum and maximum (whiskers), interquartile range from 25 to 75% (box), mean (square), median (line), and outlier (x) in a box plot and as intra-individual single data points. *AAR* area-at-risk, *ANOVA* analysis of variance, *IPC* ischemic preconditioning, *I/R* ischemia/reperfusion, *LV* left ventricle

**Fig. 3:**
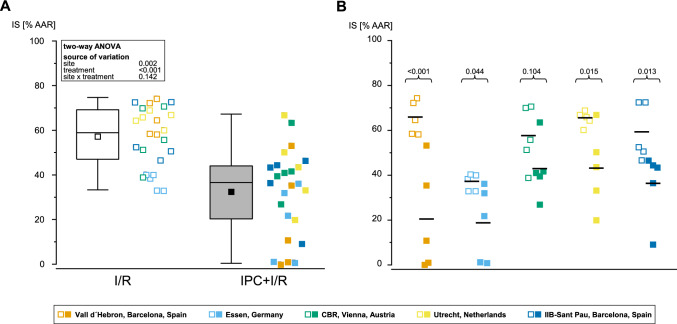
Pooled and site-specific analysis of infarct size from the 5 sites providing triphenyl-tetrazolium-chloride images. **A** Pooled analysis of IS quantified by TTC revealed a significant reduction in IS with IPC (closed squares) when compared to I/R (open squares). **B** Site-specific analysis of IS revealed significant reduction in IS with IPC in 4 sites and no reduction in IS with IPC at one site. Data are presented as minimum and maximum (whiskers), interquartile range from 25 to 75% (box), mean (square), median (line), and outlier (x) in a box plot and as intra-individual single data points. *AAR* area-at-risk, *ANOVA* analysis of variance, *IPC* ischemic preconditioning, *I/R* ischemia/reperfusion, *IS* infarct size, *TTC* triphenyl-tetrazolium-chloride

In the pooled analysis of 5 TTC sites, there were no significant differences in AAR between I/R and IPC + I/R (24 ± 7 vs. 24 ± 7 [%LV], respectively; *p* = ns; Fig. 2A). Evaluation of AAR from the individual sites revealed no significant differences among the 5 sites (Fig. 2B). In the pooled analysis of 5 TTC sites, IPC reduced IS by 44% versus I/R (57 ± 14 vs. 32 ± 19 [%AAR]; *p* < 0.001; Fig. 3A). In terms of IS from the individual sites, 4 sites achieved significant cardioprotection with IPC, whereas one site did not (Fig. 3B). When the IS was calculated as fraction of the LV, IPC reduced IS also by 43% versus I/R (14 ± 5 vs. 8 ± 5 [%LV], respectively; *p* = 0.014; Suppl. Figure 3A), and 2 sites achieved cardioprotection with IPC, whereas 3 sites did not (Suppl. Figure 3B). As mentioned above, one of the TTC sites was excluded due to the pre-defined exclusion criterion of small IS with I/R. In Suppl. Figure 2A–D, we show the pooled and individual site data of IS, including the excluded site.

### Cardioprotective efficacy of IPC assessed by CMR

**Fig. 4:**
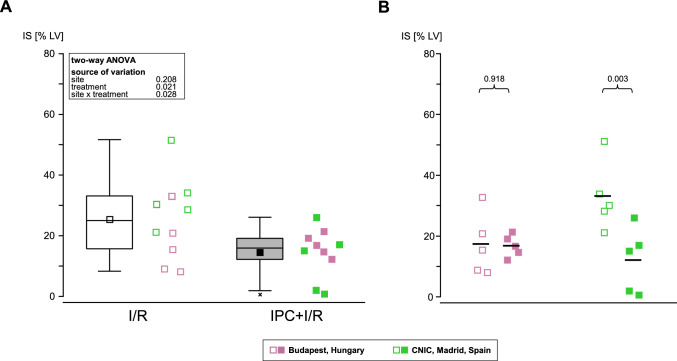
Pooled and site-specific analysis of infarct size from the 2 sites providing cardiac magnetic resonance images. **A** Pooled analysis of IS quantified by CMR revealed a significant reduction in IS with IPC (closed squares) when compared to I/R (open squares). **B** Site-specific analysis of IS quantified by CMR revealed significant reduction in IS with IPC at one site but no reduction in IS at the other site. Data are presented as minimum and maximum (whiskers), interquartile range from 25 to 75% (box), mean (square), median (line), and outlier (x) in a box plot and as intra-individual single data points. *ANOVA* analysis of variance, *CMR* cardiac magnetic resonance imaging, *IPC* ischemic preconditioning, *I/R* ischemia/reperfusion, *IS* infarct size, *LV* left ventricle

In the pooled analysis of 2 CMR sites, IPC reduced IS by 44% versus I/R (25 ± 13 vs. 14 ± 8 [%LV], respectively; *p* = 0.021; Fig. 4A). In terms of IS, one site achieved cardioprotection with IPC, the other site did not (Fig. 4B). In the pooled analysis of 2 CMR sites, there was no significant difference in AAR between I/R and IPC + I/R (Suppl. Figure 4A), although the AAR was significantly different between the 2 sites (18 ± 3 vs. 32 ± 5 [%LV]; *p* < 0.0001; Suppl. Figure 4B). In the pooled analysis of 2 CMR sites, there was no reduction in myocardial edema with IPC + I/R versus I/R (Suppl. Figure 4C), and there was no difference between the 2 sites (Suppl. Figure 4D). In the pooled analysis of the 2 CMR sites, there was no significant difference in edema between I/R and IPC + I/R (Suppl. Figure 4E), and there was no significant difference in LVEF between the 2 sites (Suppl. Figure 4F). As mentioned previously, one of the CMR sites was excluded, because it did not follow the central randomization list. In Suppl. Figure 5A–D, we show the pooled and individual site data of IS, AAR, myocardial edema, and LVEF, including the excluded site.

### Type II error for TTC and CMR data

Again, the pooled data for IS, assessed by TTC and CMR, revealed a statistically significant difference (type I error α) between IR and IPC + I/R. While the effect size was largely comparable between the TTC and CMR data (1.5 versus 1.05), the type II error (*β*) was low for the TTC data (< 1%) but not for the CMR data (27%, Table 2). Among the single sites, the effect sizes (Cohen’s *d*) ranged between 2.63 and 0.08, while the type II error for single sites on the statistical comparison of I/R vs. IPC + I/R with statistical significance ranged between 2 and 29% (Table 2).

**Table 2:** Type I error, effect size, type II error, and the estimated total *n* value per site and for the pooled data from those sites which assessed IS by TTC and CMR

Participating sites	Type I error (*α*)	Effect size (Cohen’s *d*)	Type II error (*β*)	Estimated total *N* value for 1-*β* > 0.9
Vall d´Hebron, Barcelona, Spain	< 0.001	2.62	0.0017	8
Essen, Germany	0.044	1.53	0.2867	18
CBR, Vienna, Austria	0.104	1.11	–/–	30
Utrecht, The Netherlands	0.015	1.76	0.1861	14
IIB-Sant Pau, Barcelona, Spain	0.013	1.63	0.2400	16
**Pooled data IS assessed by TTC**	** < 0.001**	**1.50**	**0.0002**	**18**
Budapest, Hungary	0.918	0.08	–/–	5080
CNIC, Madrid, Spain	0.003	2.10	0.0835	10
**Pooled data IS assessed by CMR**	**0.021**	**1.05**	**0.2727**	**34**

*CBR* Center for Basic Research, *CNIC* Centro Nacional de Investigaciones Cardiovasculares Carlos III, *CMR* cardiac magnetic resonance imaging, *IIB-Sant Pau* Sant Pau Biomedical Research Institute, *IS* infarct size, *TTC* triphenyl-tetrazolium-chloride

## Discussion

In the present study, we have established a pig AMI multicenter network for evaluating the potential efficacy of cardioprotective interventions using centralized randomization and centralized blinded core laboratory analysis of IS by both TTC (the gold-standard method for ex vivo IS quantification in pre-clinical models) and CMR (the gold-standard clinical method for in vivo IS quantification). Using IPC as a positive control, IS was reduced in our pooled data analysis, thereby validating our pig AMI multicenter network.

The establishment of the IMPACT pig AMI multicenter network operated in parallel to our IMPACT small animal AMI multicenter network [20] and completes the translational pathway highlighted in the IMPACT criteria for improving the translation of cardioprotective interventions for patient benefit [45]. According to the step-by-step IMPACT criteria, once the efficacy of the cardioprotective intervention has been demonstrated in a pig AMI study at a single site, one should consider evaluating the cardioprotective intervention in a multicenter pig AMI study using centralized randomization and centralized blinded core laboratory analysis of IS to demonstrate the robustness of the finding from the single original site. In this regard, the IMPACT pig AMI network, which has been established in the present study, would be suitable for this purpose.

Again, a multicenter network for undertaking in vivo pre-clinical evaluation of novel cardioprotective therapies was first demonstrated by the CAESAR research network where 3 sites performed AMI in mice, rabbits, and pigs, and IPC was reported to reduce IS [35]. CIBER-CLAP also used IPC as positive control [50]; however, results of this network have not yet been published. Of importance, our IMPACT pig AMI multicenter network differs from the CAESAR consortium and CIBER-CLAP in several important aspects. With our pan-European multicenter network, in which ten sites agreed to participate, we overcome all logistical challenges associated with implementation of a large multisite pre-clinical study across national borders. Whereas the two prior networks enforced a strict approach to standardization of study procedures (including species, the IPC and I/R protocols, anesthetics and analgesics, and histological sample preparation) which for the CAESAR consortium extended to the animal husbandry protocols (e.g., with the animal diets and housing conditions), our IMPACT network did not. We did not standardize the pig breeds, age, sex, diets, and housing conditions, the study protocol, the analgesia and anesthesia, the use of anti-arrhythmic drugs, the experimental preparation (open versus closed chest), and the IPC protocol. Of course, these different conditions and the variability between sites prevented us from comparing IS, its reduction by IPC, and the statistical power between sites. Despite these wide differences in study conditions across the sites, the pooled and most site-specific analyses demonstrated cardioprotection with IPC when compared to I/R, underscoring the robustness of IPC-induced cardioprotection in pig AMI models. Although this lack of homogeneity in experimental procedures has often been cited as one of the reasons for the lack of reproducibility in animal studies [51, 54], our results suggest that in a multicenter network, non-standardization of study protocols may be preferable and more accurately reflect the real-world setting of AMI patients. More pragmatically, this approach also allows each laboratory to use local study protocols established at that particular site. Although we did not standardize the study conditions, we performed an intensive and strict QC to standardize infarct images for both TTC and CMR analyses by central core laboratories to ensure the accurate and high-quality quantification of IS. This process was challenging, and some centers had to perform additional experiments to optimize the preparation of hearts for TTC. We excluded one site due to a low IS with I/R, and this small IS may explain why IPC did not reduce IS further at this site (Suppl. Figure 2D). To follow up, this site undertook a new series of pig AMI studies, and this time, the site did not use pre-medication with the anti-arrhythmic drug amiodarone, and found an increase in IS with I/R and reduced IS with IPC when compared to I/R (Suppl. Figure 6B). The use of amiodarone has been reported to reduce IS per se [11].

Since the type II error for the IS data of the individual sites was up to 29%, statements about the reduction in IS in some sites but not in others must be treated with great caution. Nevertheless, IS was reduced in tendency at all sites, and for 4 of 5 sites which used TTC for IS assessment (as proportion of AAR), this reduction was statistically significant. However, when IS was calculated as a proportion of LV, IPC reduced IS at only 2 of 5 sites with statistical significance. The most robust endpoint of experimental cardioprotection studies is the reduction in IS as a proportion of AAR [8]. In contrast, in clinical trials using imaging techniques, IS is often calculated as a proportion of LV because of an inability to accurately measure the AAR [34]. Also, the proportion of the LV that is salvaged from infarction is probably more important for the long-term prognosis of AMI patients [22].

One site failed to observe any IS reduction with IPC assessed by CMR; remarkably, there was not even a trend toward an IS reduction. These results illustrate the limitations in reproducibility of cardioprotection studies despite strict QC and the reasons for this failure are not clear. For this CMR site, AAR and IS with I/R were smaller than at the other sites (Suppl. Figure 4B, 5D). Myocardial damage is primarily determined by the AAR, and again, the smaller the damage, the smaller the possible protection that can be induced [29]. For this reason, we agreed before the start of the study that a small IS as fraction of the AAR would be an exclusion criterion for the TTC sites. Since the gold standard for quantification of AAR by imaging techniques is contrast computed tomography, which was not available at all sites, and the BARI score is less accurate [34], this exclusion criterion was not used for the CMR sites. This aspect should be considered more carefully in future studies. However, differences in animal strains, the I/R protocol, the anesthesia or analgesia regimens or individual genetically determined, and primordial non-responsiveness of the myocardium to cardioprotection [28, 33, 52, 61] could also be of importance here. The pig breed at this specific site may have had a genetic variant that made it more resistant to IPC, and indeed, in the previous studies, this site had already published neutral results on cardioprotective maneuvers in pigs [3]. The use of inbred pig strains definitely does not reflect the human situation, and genetic heterogeneity by the use of different pig breeds in this multicenter network may thus better reflect human reality. In fact, the robust protection by IPC reflected through the published literature may not correspond to reality, neutral studies are definitively underreported in pre-clinical studies [53].

**Fig. 5:**
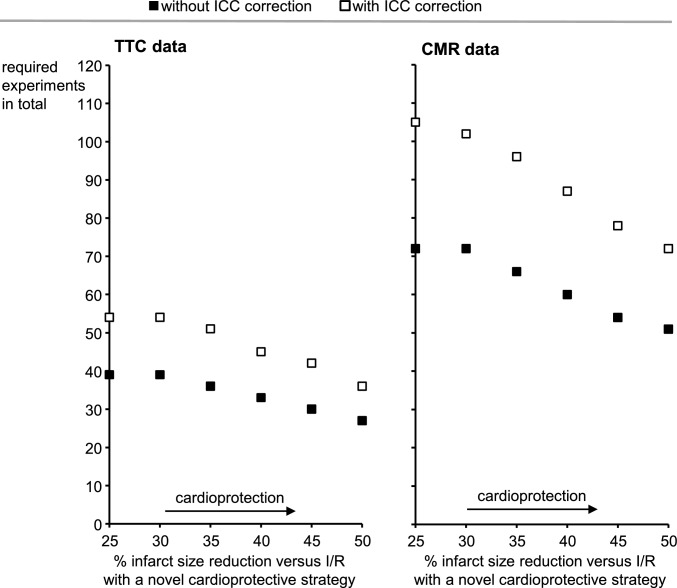
Total number of animals required for testing a novel cardioprotective strategy when using our IMPACT network with infarct size quantified with TTC (**A**) or CMR (**B**). The *Y*-axis depicts the total number of animals and the *X*-axis depicts the relative reduction in infarct size. Calculations are based on the observed IMPACT network IS data with I/R and IPC + I/R and *α* ≤ 0.05 with statistical power 1-*β* of ≥ 0.9. *AAR* area-at-risk, *CMR* cardiac magnetic resonance imaging, *ICC* intraclass correlation coefficient, *IPC* ischemic preconditioning, *I/R* ischemia/reperfusion, *IS* infarct size, *LV* left ventricle, *TTC* triphenyl-tetrazolium-chloride

To aid the design of future pig AMI cardioprotection studies in terms of prospective calculation of required sample size, we used the present data to estimate the effect size for a hypothetical experimental design with 3 groups (I/R, IPC + I/R, and a novel cardioprotective strategy + I/R). We additionally assumed that the IS reduction induced by the novel cardioprotective strategy is only of half the magnitude of that by IPC. A recurrent inclusion of an IPC + I/R group as a positive control appears reasonable if not mandatory, since some sites were unable to demonstrate a reduction in IS with IPC using their experimental setup, and it must be assumed that other cardioprotective strategies also may fail there. IPC can then be used not only as a positive control, but also for QC and scaling of results against the relatively large data variability between sites. Using the tool G-Power 3.1, we estimated the effect size to compute the required *n* value for an ANOVA analysis (fixed effects, one-way) given *α* = 0.05, power 1-*β* = 0.9, as recommended previously [8]. When using the pooled TTC data, effect size was *f* = 0.7, requiring a total number of 39 experiments (≙ *n* = 13 per group, to be covered by the participating sites); when the pooled CMR data were used, the calculated effect size was *f* = 0.48, requiring a total number of 75 experiments (≙ *n* = 25 per group). However, these calculations do not take into account that there are obvious differences in IS and IS reduction by IPC between the sites. We therefore calculated the ICC (one-way random effects, absolute agreement, and single rater per measurement) [41] and corrected the previously determined effect size by 1-ICC [60]. As ICC of I/R was substantially larger than that of IPC + I/R (0.175 vs. 0.047), we used only the ICC of I/R for the effect size correction. This correction increased the total required number of experiments from 39 to 54 when using the TTC data and from 75 to 111 for the CMR data. However, when using the TTC data from each single site for the same hypothetical design above, the total number of experiments required is considerably lower with *n* = 15, 30, 33, or 36 (except for *n* = 66 for that site which did not show significant IS reduction with IPC). To provide potential users of our current network (e.g., pharmaceutical companies) with an understanding of the resources and costs associated with conducting cardioprotection studies, we have developed a diagram (Fig. 5) illustrating the required number of animals based on the observed variabilities in the IMPACT network for a given intervention and *α* ≤ 0.05 and a power 1-*β* of ≥ 0.9 for the target effect size, both with and without the ICC correction. This estimate is based on the IPC + I/R versus I/R IS from this IMPACT network. Prior to testing a novel cardioprotective strategy, however, this comparison must be repeated to obtain an up-to-date scaling for an established cardioprotective measure.

The current multicenter approach revealed challenges of QC, a high variability of results, and the need for a high number of experiments but also revealed that such multicenter approach resembling a real-life clinical trial can identify significant cardioprotection, while individual studies may show neutral outcomes. Given these challenges, it remains open whether any company will be interested in a network such as ours.

## Study limitations

Our strategic decision not to use highly standardized settings across the sites has two implications. On the one hand, this approach aims to recapitulate the varied conditions found in the clinical setting and reduces errors in protocol adherence by simplifying local study logistics. On the other hand, using pigs of different breeds, age, and sex, and the different experimental protocols, including reperfusion time and TTC staining procedures will also increase variability in IS as the primary outcome parameter and accordingly the number of animals needed for robust results. However, in our rather liberally designed IMPACT approach, the observed coefficient of variance (CV) of IS (as % AAR by TTC) was very similar to that observed in the CAESAR network [35] using the same endpoint but with a high standardization of all experimental settings across the three sites (CV with I/R: 24% IMPACT and 25% CAESAR network; with IPC + I/R: 60% IMPACT and 52% for CAESAR research network).

In the present study, we focused only on IS as the primary endpoint, but as outlined in the IMPACT criteria, it is increasingly recognized that coronary microvascular injury is a manifestation of AMI and therefore an additional target for cardioprotection [17, 23, 28]. Both histochemistry, e.g., with use of thioflavin staining [38], and CMR [64] allow for the study of microvascular damage (microvascular obstruction, hemorrhage), and it should therefore be included in the design of future studies as a secondary outcome. Only two sites quantified IS using the clinical gold standard of CMR, and future trials should ensure more laboratories with CMR capabilities [28].

Notably, in the present study, the effect size for the CMR data was smaller, and the type II error was remarkably higher than for the TTC data. However, the CMR data on IS reduction by IPC came from only two sites, and for one site, there was not even a trend toward an IS reduction. Again, this site had already failed to reproduce otherwise established cardioprotective maneuvers in pigs [3]. IS reduction in humans where CMR is the gold standard may be more difficult and require larger sample sizes than with TTC in animals. Nevertheless, we are confident that our current data do not argue against the use of CMR in cardioprotection studies.

With I/R, IS as fraction of LV was smaller, when assessed by TTC than by CMR (Suppl. Figure 3A vs. Figure 4A). In the literature, however, the overall assessment of IS by TTC and CMR appears to be comparable [36], although there are notable differences in methodology. TTC stains myocardium in the presence of intact dehydrogenase enzyme systems red, while dead tissue without intact dehydrogenase enzyme systems remains unstained [15]. The late gadolinium enhancement sequence correlates with the amount of contrast retained in the extracellular myocardial space, which may be distributed between clusters of cardiomyocytes that are not completely dead [34]. In addition, CMR typically overestimates IS when edema and inflammation have not fully resolved after myocardial infarction. To avoid this first wave of dynamic post-reperfusion edema, days 5–7 of reperfusion are used as the gold standard for quantification of IS [34]. On the other hand, TTC staining has only been validated for reperfusion times of minutes/hours [15]. Given these methodological differences, it is reasonable to assume that there may be a discrepancy in measured IS between the two techniques. However, there are no data available comparing IS quantification by TTC and CMR or even validating it with histologic staining in the reperfusion time frame used here.

We used juvenile/young (except for one site) healthy animals free of comorbidities and comedications which are known to confound cardioprotection [7, 13, 37]. Although challenging, testing of cardioprotective interventions in a pig model which is closer to the human situation would be ideal to improve translation and it is usually required by the regulatory authorities in case of drug or medical device development. There are pig models developing hypercholesterolemia [62] after special diet or hypercholesterolemia in combination with diabetes mellitus (induced via streptozotocin) [58]. However, none of these models develop a full metabolic syndrome as seen in patients. The feral pig breed Ossabaw minipigs develop the full metabolic syndrome, including obesity, glucose intolerance, insulin resistance, hypertension, and dyslipidemia after consumption of a hypercaloric and atherogenic diet. Ossabaw minipigs are characterized by vascular dysfunction [12], and develop diffuse coronary atherosclerosis, including plaque instability and subsequent thrombosis on a polygenic background [56, 65]. Unfortunately, however, these minipigs do not respond to IPC with IS reduction [39]. There are novel, clinically relevant experimental strategies in pigs, such as P2Y12 inhibitor preloading as used in AMI patients [63], which we also did not consider except for 1 site.

We did not register our study on platforms such as the recently established PCT (https://preclinicaltrials.eu/), which corresponds to established clinical registries. Prior registration of the study hypothesis and protocol not only creates transparency throughout the scientific community, but also raises awareness of bias reduction measures such as randomization and blinding. Finally, registration of pre-clinical studies also increases the comparability with clinical studies [59].

Another minor limitation of the present study relates to the lack of utilization of a unified database (e.g., REDCap), as seen in other types of multi-center trials in human patients. Although both core laboratories performed data collection for TTC and CMR studies, the use of unified databases allows for validation and complete auditing of all data collection, exportation, and analysis in a centralized manner. Obviously, this limitation does not jeopardize or penalize the results of the current study, but from an organizational and methodological standpoint, it would be beneficial to include them whenever possible.

Finally, no external advisors were consulted in the design of the present network study, as is usual in clinical multi-center trials. During the QC process, it became obvious that the involvement of such advisors would further improve the quality of our multicenter networks.

## Conclusion

**Fig. 6:**
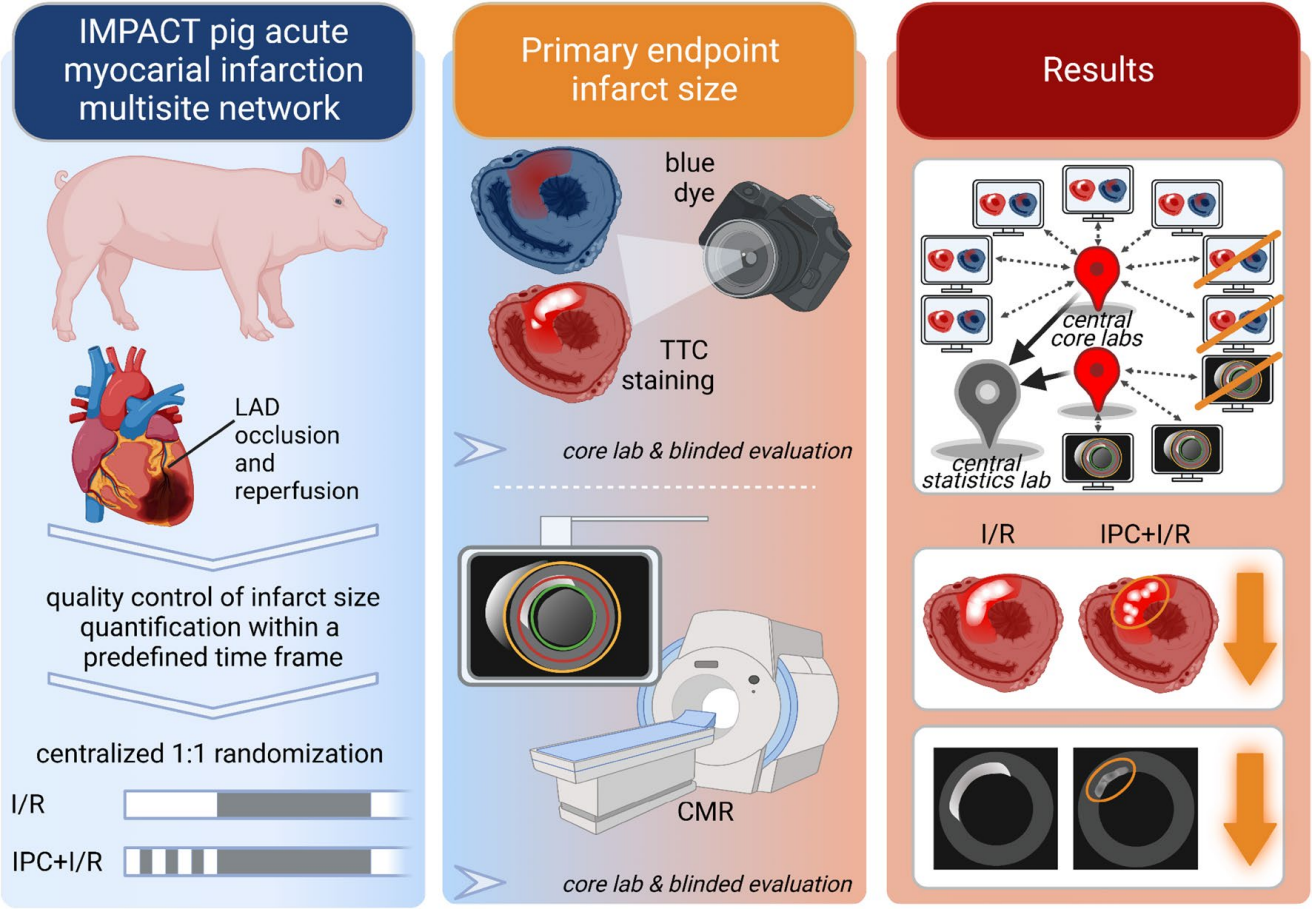
The multicenter network evaluated the cardioprotective effect of IPC as the decrease in infarct size measured ex vivo by TTC and in vivo by CMR. *CMR* cardiac magnetic resonance imaging, *IPC* ischemic preconditioning, *I/R* ischemia/reperfusion, *LAD* left anterior descending coronary artery, *TTC* triphenyl-tetrazolium-chloride. Created with BioRender.com

Despite the fact that not all sites demonstrated cardioprotection with IPC, there was a significant reduction in IS with IPC in the pooled analyses, emphasizing the high value of the multicenter network approach in this field. Thus, we here have established and validated a new IMPACT pig AMI multicenter European network with centralized randomization and central QC as well as core blinded IS analysis by TTC and CMR (Fig. 6). This pig AMI network can be used to improve the rigor and robustness of pre-clinical studies evaluating the efficacy of cardioprotective interventions and may increase the likelihood of translation of cardioprotection for patient benefit.

## Supplementary Information

Below is the link to the electronic supplementary material.

Supplementary file1 (PDF 952 KB)

Supplementary file2 (XLSX 63 KB)

## Data Availability

Data will be made available upon reasonable request.
