# Supplementary material for: The IMproving Preclinical Assessment of Cardioprotective Therapies (IMPACT): multicenter pig study on the effect of ischemic preconditioning
Source: Basic Res Cardiol. 2024 Oct 18;119(6):893–909. doi: 10.1007/s00395-024-01083-9 (PMC11628588; doi:10.1007/s00395-024-01083-9)
Supplement: Supplementary file 1 — Supplementary file1 (PDF 952 KB) [file 395_2024_1083_MOESM1_ESM.pdf]

Supplemental methods

and

supplemental tables and figures

The IMproving Preclinical Assessment of Cardioprotective Therapies (IMPACT) – multicenter pig study on the effect of ischemic preconditioning

Petra Kleinbongard*; Carlos Galán Arriola*; Lina Badimon; Veronica Crisostomo; Zoltan Giricz; Mariann Gyöngyösi; Gerd Heusch; Borja Ibanez; Attila Kiss; Dominique de Kleijn; Bruno Podesser; Rafael Ramírez Carracedo; Antonio Rodríguez-Sinovas; Marisol Ruiz-Meana; Francisco Sanchez Margallo; Gemma Vilahur; José Luis Zamorano; Carlos Zaragoza; Peter Ferdinandy**; Derek J. Hausenloy**

**shared co-first authors*
***shared senior authors*

Supplemental methods cardiac magnetic resonance imaging (CMR)

Sites: Budapest, Hungary and Dep Med II, Vienna, Austria

Baseline CMR scans were performed 1-4 days before myocardial infarction and scans subsequently repeated at post-infarction follow-up before sacrifice. All studies were performed with a 1.5 T clinical scanner (Avanto, Siemens Healthcare, Best, the Netherlands) using a phased array coil and a vector electrocardiogram (ECG) system to assess the cardiac function (ejection fraction, cardiac mass and volumes). CMR images were acquired using a retrospectively ECG-gated, steady-state free precession CMR technique in short-axis and long-axis views of the heart using 1.2 ms echo time, 40 ms repetition time, 50-degree flip angle, 300 mm field-of-view, 8 mm slice thickness, and 256x256 image matrix. CMR recordings were obtained in apical to basal direction including both the apex and base.

CMR scans were performed at 7 days post MI of each animal according to assess cardiac dimensions as well as infarct size and edema appearance. CMR scans were performed essentially as described for baseline CMR. An intravenous bolus of contrast agent (0.05 mmol/kg gadobutrol, [Gadavist, Bayer]) at a rate of 4 ml/sec) was infused using a manual injector. Prior to CMR, 0.6-1.5 mg/kg Tracrium was administered and ventilation were maintained with mechanical ventilation. Delayed enhancement images were obtained using an inversion recovery-prepared, gradient-echo sequence. Short axis and long axis images were obtained 8 to 15 min after gadolinium injection and evaluated by the 6 standard deviations method.

Site: CNIC, Madrid, Spain

All baseline CMR scans were performed the same day than the myocardial infarction and subsequently repeated at 6 days post-infarction follow-up time before sacrifice. All scans were performed with a 3T clinical scanner (Philips Healthcare, Best, the Netherlands) equipped with a 32-element phased-array cardiac coil and ECG system to assess thee cardiac function (ejection fraction, cardiac mass and volumes). Cine CMR images were acquired using a retrospectively ECG-gated, steady-state free precession cine CMR technique in short-axis and long-axis views of the heart using 1.4 ms echo time, 2.8 ms repetition time, 45-degree flip angle, 280 mm field-of-view, 8 mm slice thickness, and 450x450 image matrix. CMR recordings were obtained in apical to basal direction including both the apex and base.

CMR scans were performed at 6 days post MI of each animal according to assess cardiac dimensions as well as infarct size, edema and MVO appearance. Native cardiac MRI were performed essentially as described for baseline. An intravenous bolus of contrast agent (0.2 mmol/kg gadopentate dimeglumine, [Clariscan, Opakim A.S.]) was infused using an automatic injector. Delayed enhancement images were obtained using an 3D inversion recovery-prepared, gradient-echo sequence. Images were obtained 15 minutes after gadolinium injection and evaluated by the 6 standard deviations method.

Supplemental Table 1: Quality control criteria for the two techniques used in the assessment of infarct size (triphenyl-tetrazolium-chloride and cardiac magnetic resonance imaging).

**Checklist for quality control of the triphenyl-tetrazolium-chloride images**
1. Ruler present as reference scale<br>2. Labels present to indicate the experiment and slice number<br>3. Slice completely immersed in saline to avoid torsion<br>4. No reflections from light sources<br>5. Regions of interest clearly visible<br>6. Image sharp and in focus<br>7. Area-at-risk delineated<br>8. Triphenyl-tetrazolium-chloride negative areas delineated
**Cardiac magnetic resonance imaging checklist for quality control**
1. All sequences correctly labeled<br>2. CINE sequences:<br>• free of motion artifacts<br>• sufficient phases to correctly set the end-systolic/diastolic phases<br>• sufficient slices covering the whole left ventricle from base to apex<br>• sufficient contrast between the myocardium and the blood pool<br>3. Late gadolinium Enhancement (LGE)/edema sequences:<br>• free of motion artifacts<br>• Gadolinium sufficiently washed-out and the extracellular volume (ECV) expansion-LGE deposition enough contrasted<br>• sufficient slices covering the whole left ventricle from base to apex

A

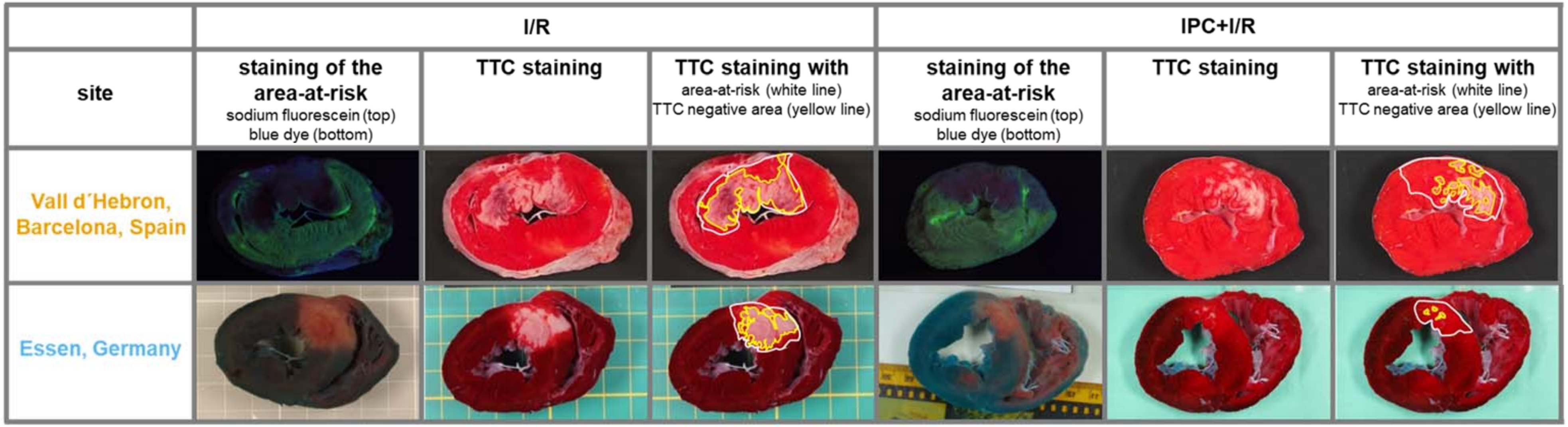

Supplemental Figure 1: Representative images for infarct size quantification via TTC (A) and CMR (B).
CMR: cardiac magnetic resonance imaging; IPC: ischemic preconditioning; I/R: ischemia/reperfusion; TTC: triphenyl-tetrazolium-chloride

B

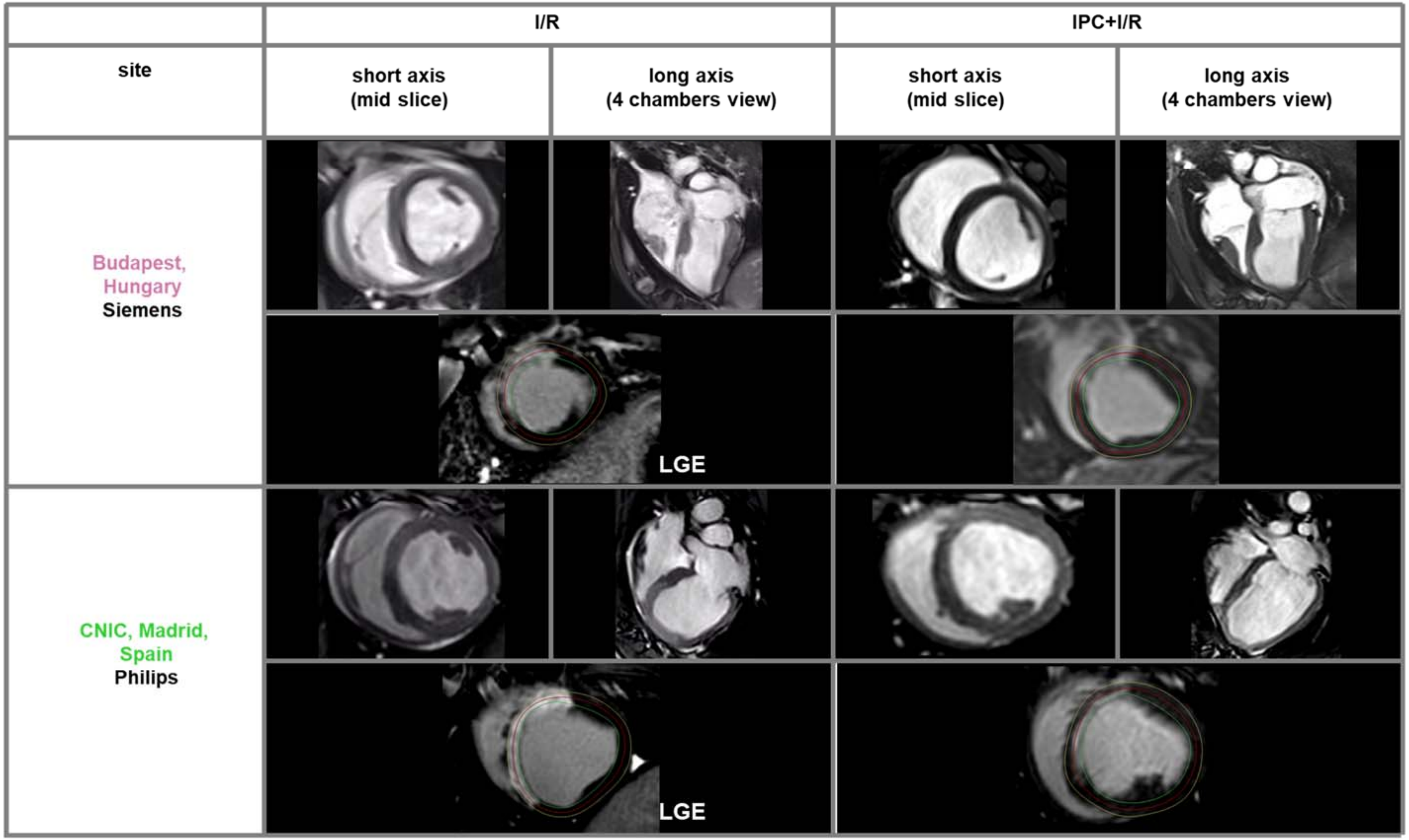

Supplemental Figure 1 continued

Supplemental Table 2: List of fibrillation periods and applied defibrillation shocks as mean±standard deviations (SD) per site and protocol.

Participating sites	Vall d´Hebron, Barcelona, Spain		Essen, Germany		CBR, Vienna, Austria		Utrecht, Netherlands		IIB-Sant Pau, Barcelona, Spain		CCMIJU, Cáceres, Spain		Dep Med II, Vienna, Austria		CCNIC, Madrid, Spain		Budapest, Hungary	
**I/R** (n=5 per site)	fibrillation periods	applied defibrillation shocks	fibrillation periods	applied defibrillation shocks	fibrillation periods	applied defibrillation shocks	fibrillation periods	applied defibrillation shocks	fibrillation periods	applied defibrillation shocks	fibrillation periods	applied defibrillation shocks	fibrillation periods	applied defibrillation shocks	fibrillation periods	applied defibrillation shocks	fibrillation periods	applied defibrillation shocks
during ischemia	0.2±0.5	0.4±0.9	2.0±2.8	3.6±5.0	0.2	n.r.	1.2±1.3	6.8±7.4	2±0	2±0	2.0±2.9	1.6±2.1	0	0	4±0	4±0	1.3±0.5	0.4±0.5
during reperfusion	0	0	0.2±0.4	0.4±0.9	0	n.r.	0	0	0	0	0	0	0	0	0	0	0.8±0.6	0.4±0.5
**IPC+I/R** (n=5 per site)	fibrillation periods	applied defibrillation shocks	fibrillation periods	applied defibrillation shocks	fibrillation periods	applied defibrillation shocks	fibrillation periods	applied defibrillation shocks	fibrillation periods	applied defibrillation shocks	fibrillation periods	applied defibrillation shocks	fibrillation periods	applied defibrillation shocks	fibrillation periods	applied defibrillation shocks	fibrillation periods	applied defibrillation shocks
during IPC	0	0	0	0	0	n.r.	0	0	0	0	0	0	0	0	0	0	0.1±1.0	0
during ischemia	0	0	3.2±4.5	6.0±9.2	0	n.r.	2.8±1.7	8.4±7.3	3±0	3±0	1.4±1.1	1.4±1.1	0.2±0.4	0.4±0.9	6±0	6±0	1.6±1.1	0.6±0.7
during reperfusion	1.6±2.0	2.4±3.7	0.4±0.9	1.0±2.2	0.4	n.r.	0	0	0	0	0	0	0	0	0	0	1.4±1.0	0

IPC: ischemic preconditioning; I/R: ischemia/reperfusion; n.r.: not recorded

Supplemental Table 3: List of excluded pigs after passing initial quality control (QC) and assigning pigs to ischemia/reperfusion (I/R) and ischemic preconditioning (IPC) according to the central randomization list. Details of all site-specific exclusion criteria can be found in “Suppl. Data sheet experimental design”.

**Participating sites**	**Exclusion due to pre-defined criteria**	**Death during experimental procedure**	
		during ischemia	during reperfusion
Vall d´Hebron, Barcelona, Spain	-	-	-
Essen, Germany	n=4 via RMBF	-	-
CBR, Vienna, Austria	n=3 via QC	n=2 I/R VF	
Utrecht, Netherlands	-	n=2 I/R VF	-
IIB-Sant Pau, Barcelona, Spain	n=2 via QC	-	-
CCMIJU, Cáceres, Spain	n=2 via QC	-	n=1 IPC+I/R VF
CNIC, Madrid, Spain	-	n=2 I/R VF	-
Budapest, Hungary	n=2 via balloon rupture and baseline bradycardia and hypotension	n=1 IPC+I/R VF	

IPC: ischemic preconditioning; I/R: ischemia/reperfusion; RMBF: regional myocardial blood flow in the area-at-risk; VF: non-terminable ventricular fibrillation

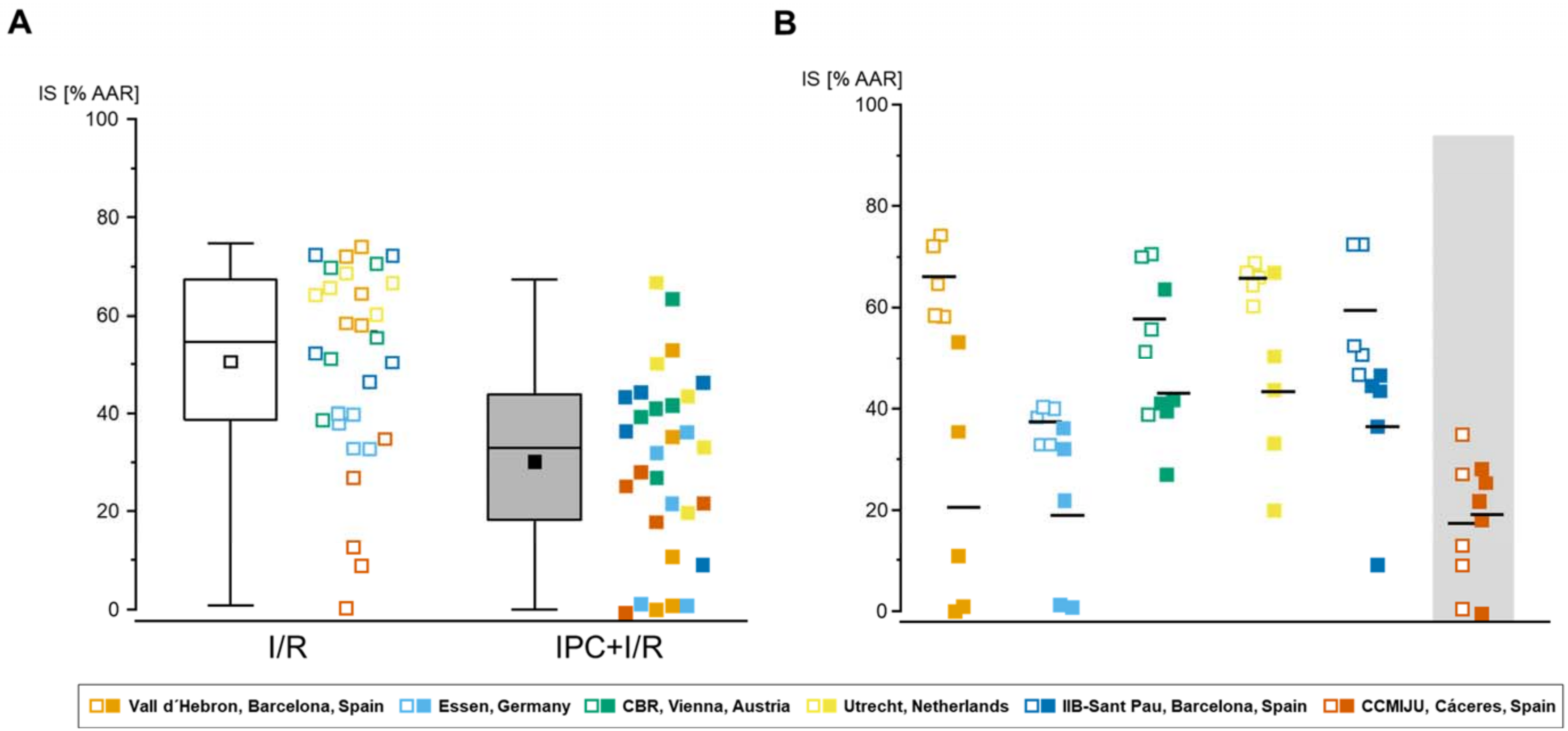

Supplemental Figure 2: Pooled and site-specific analysis of infarct size from the all sites providing triphenyl-tetrazolium-chloride images (with site excluded due to a small infarct size in the control group according to the predefined criteria, data of this site are highlighted in gray)

(A) Pooled analysis of IS revealed a significant reduction in IS with IPC (closed squares) when compared to plain I/R (open squares). **(B)** Site-specific analysis of IS. **(C, D)** Pooled and site-specific analysis of infarct size - calculated as a fraction of the left ventricle.

Data are presented as minimum and maximum (whiskers), interquartile range from 25 to 75% (box), mean (square), median (line), and outlier (x) in a box plot and as intra-individual single data points. These data were not statistically analyzed, as data from an excluded center are presented.

AAR: area-at-risk; IPC: ischemic preconditioning; I/R: ischemia/reperfusion; IS: infarct size; LV: left ventricle; TTC: triphenyl-tetrazolium-chloride

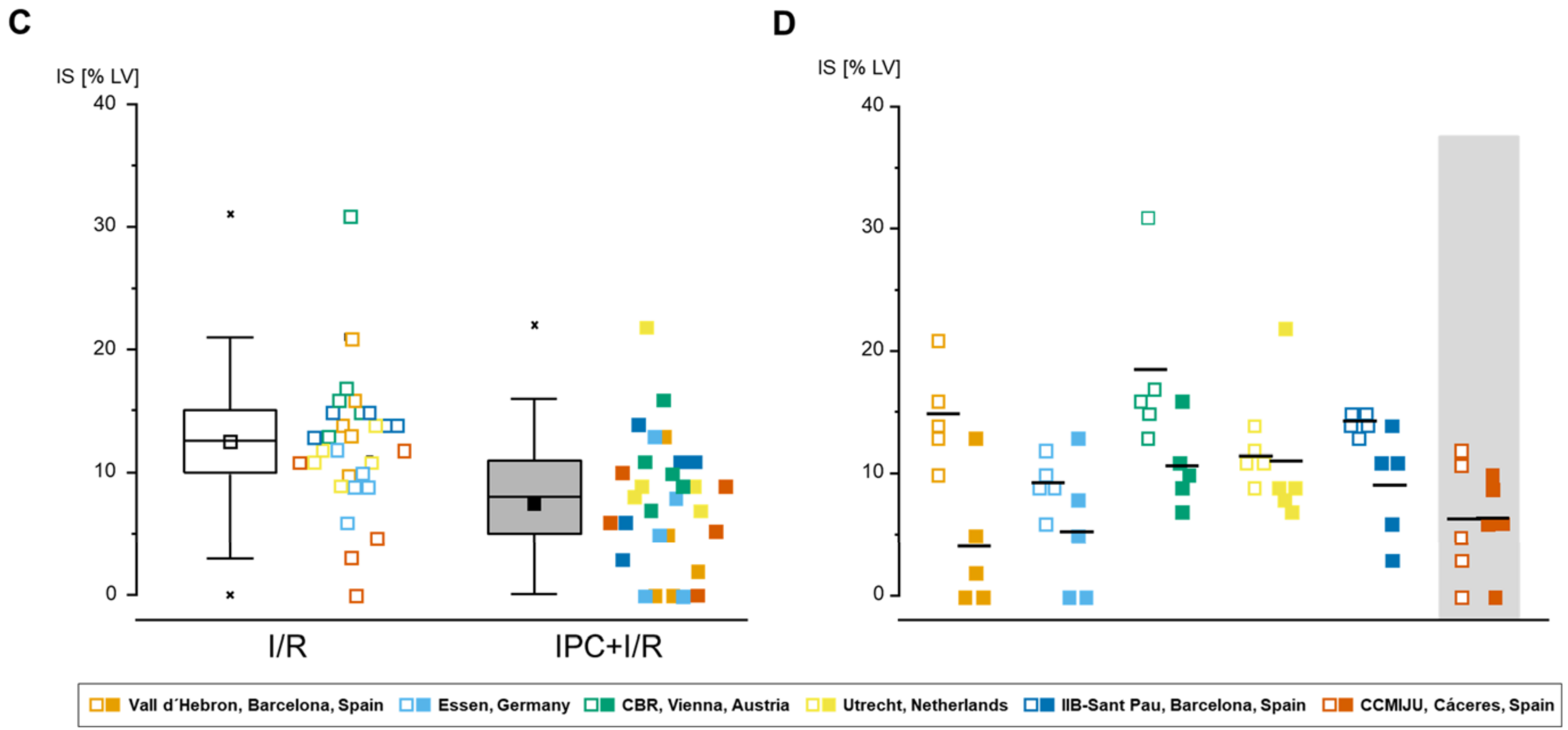

Supplemental Figure 2 continued

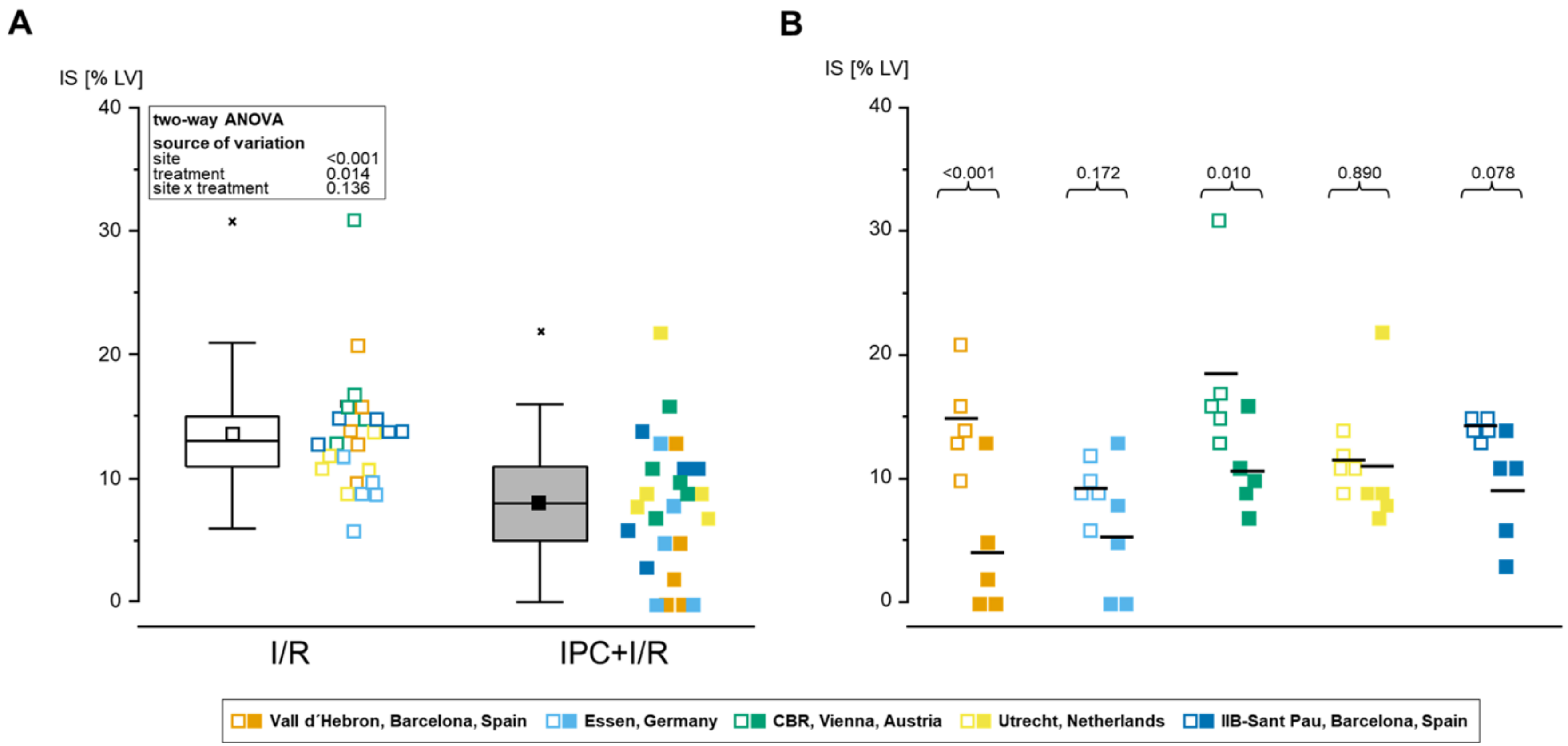

Supplemental Figure 3: Pooled and site-specific analysis of infarct size from the 5 sites providing triphenyl-tetrazolium-chloride images - calculated as a fraction of the left ventricle

(A) Pooled analysis of IS quantified by TTC revealed a significant reduction in IS with IPC (closed squares) when compared to plain I/R (open squares). **(B)** Site-specific analysis of IS revealed significant reduction in IS with IPC in 2 sites and no reduction in IS with IPC in 3 sites. Data are presented as minimum and maximum (whiskers), interquartile range from 25 to 75% (box), mean (square), median (line), and outlier (x) in a box plot and as intra-individual single data points.

ANOVA: analysis of variance; IPC: ischemic preconditioning; I/R: ischemia/reperfusion; IS: infarct size; LV: left ventricle; TTC: triphenyl-tetrazolium-chloride

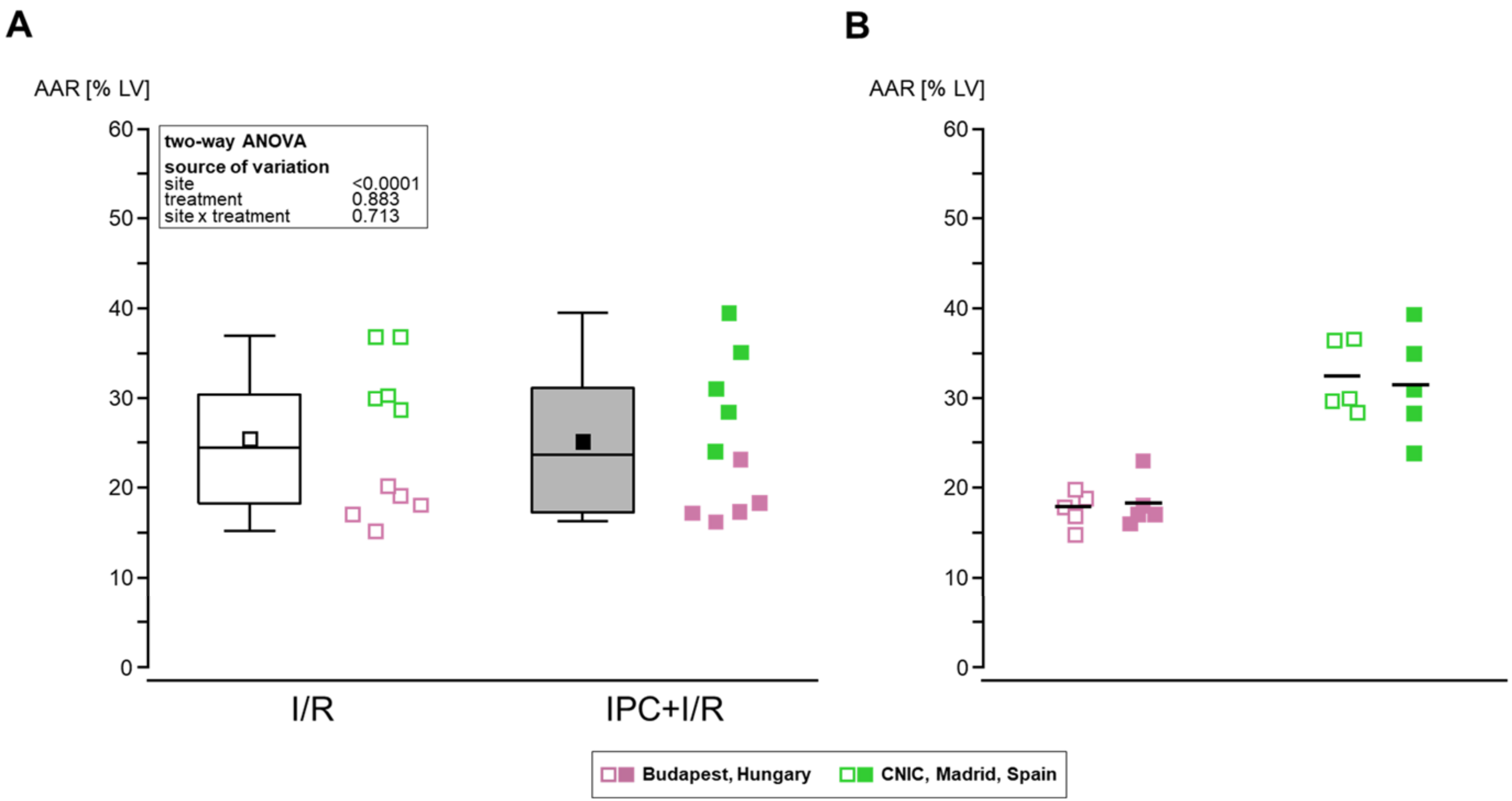

Supplemental Figure 4: Pooled and site-specific analysis of area-at-risk, myocardial edema and left ventricular ejection fraction from the 2 sites providing cardiac magnetic resonance images

(A) Pooled analysis of AAR was comparable between I/R (open squares) and IPC+I/R (closed squares). **(B)** Site-specific analysis of AAR revealed a significant difference between the sites. **(C)** Pooled analysis of myocardial edema revealed a non-significant reduction with IPC when compared to plain I/R. **(D)** Myocardial edema was comparable between the 2 sites. **(E)** Pooled analysis of LVEF was comparable between I/R and IPC+I/R. **(F)** LVEF was comparable between the 2 sites. Data are presented as minimum and maximum (whiskers), interquartile range from 25 to 75% (box), mean (square), median (line), and outlier (x) in a box plot and as intra-individual single data points.

AAR: area-at-risk; ANOVA: analysis of variance; CMR: cardiac magnetic resonance imaging; IPC: ischemic preconditioning; I/R: ischemia/reperfusion; LV: left ventricle; LVEF: left ventricular ejection fraction

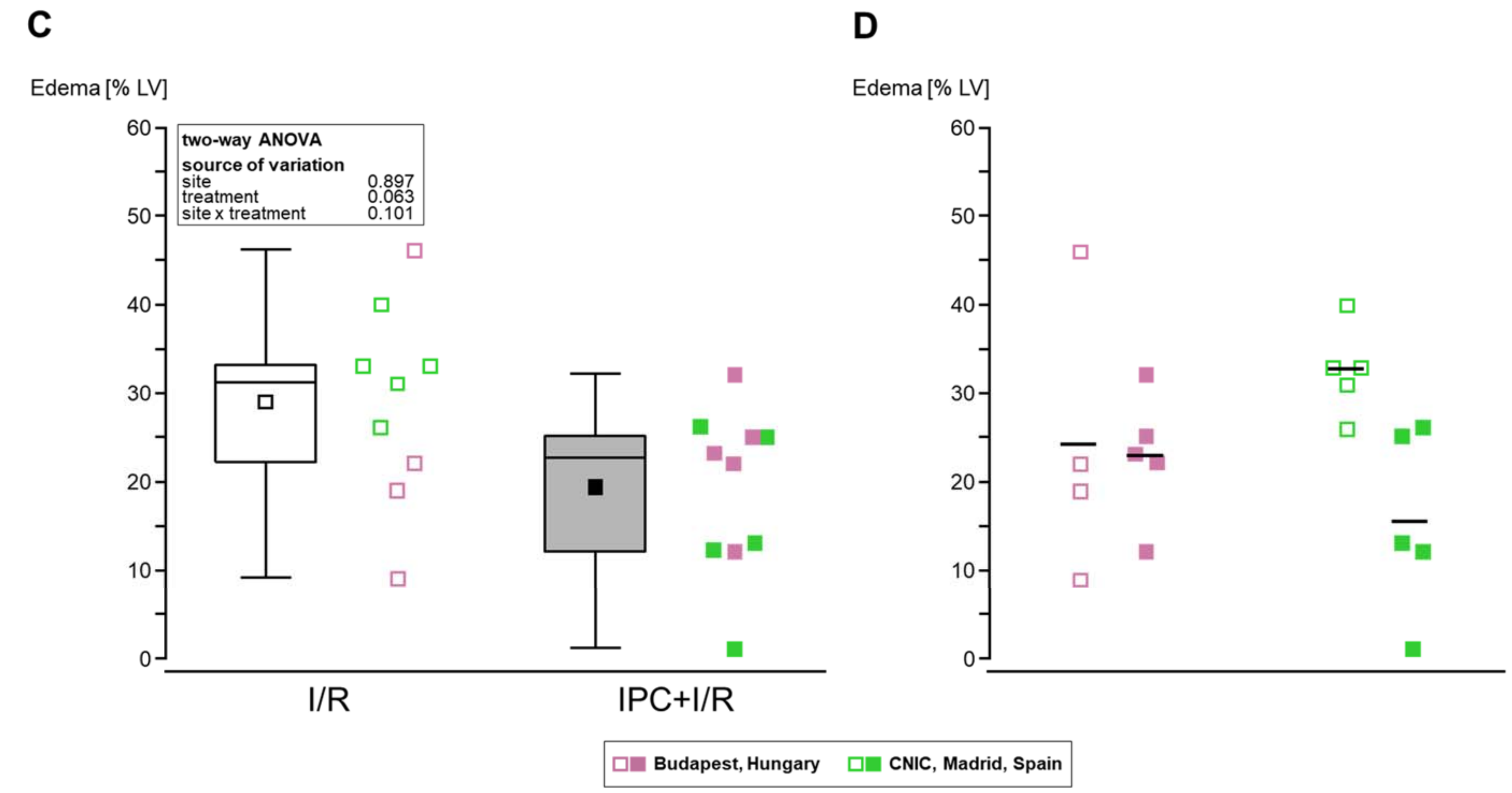

Supplemental Figure 4 continued

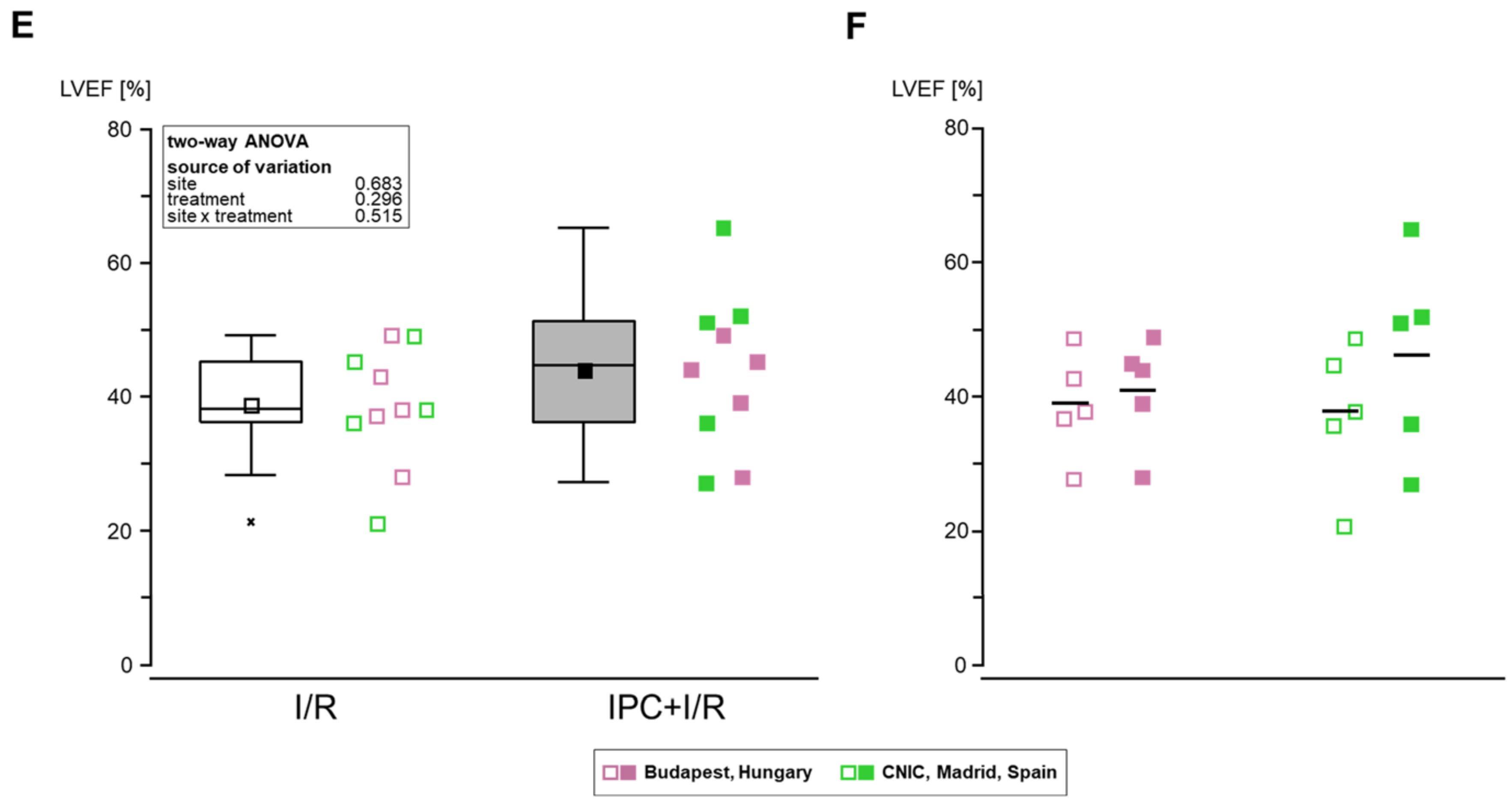

Supplemental Figure 4 continued

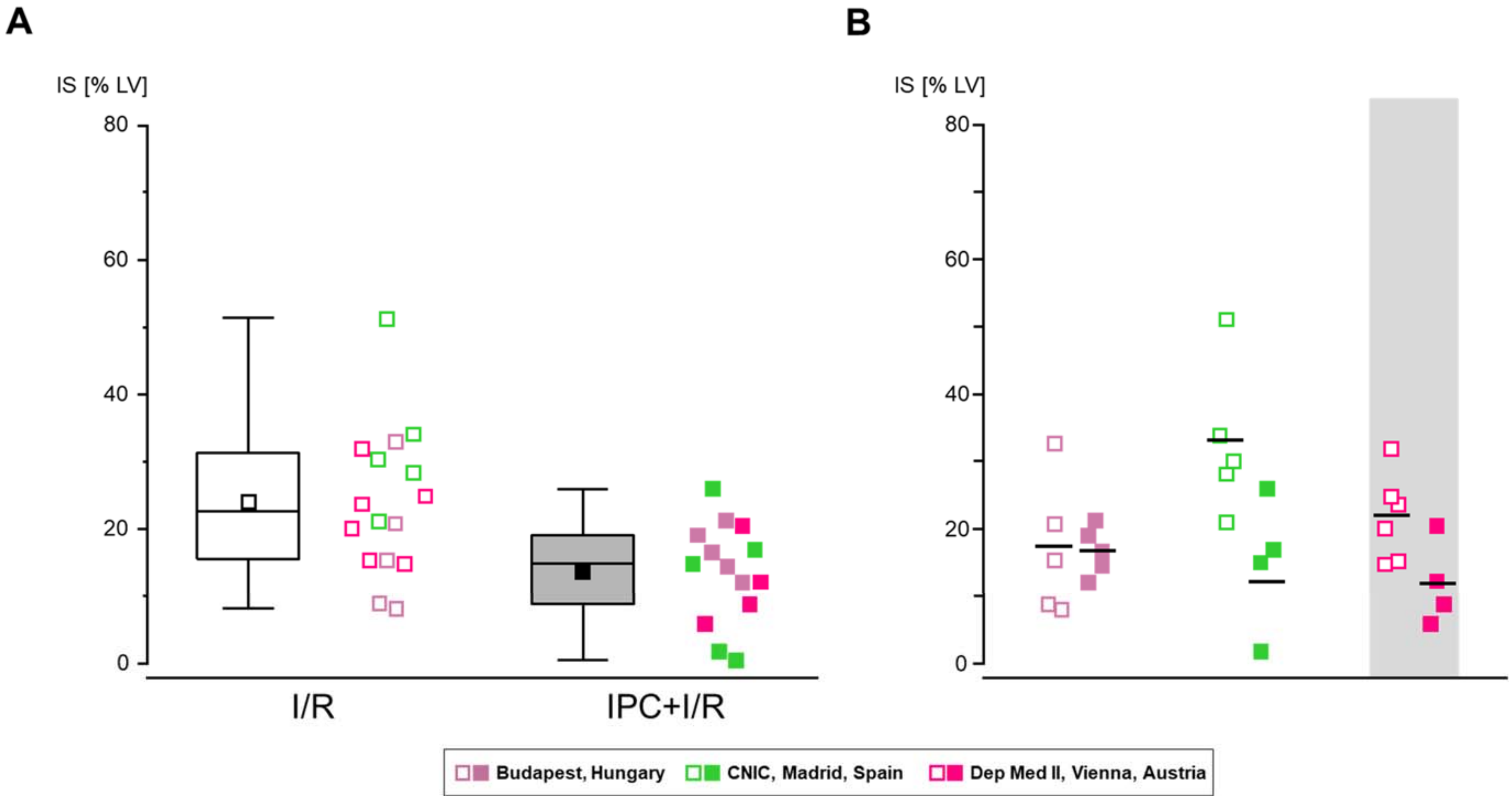

Supplemental Figure 5: Pooled and site-specific cardiac magnetic resonance imaging analysis of infarct size (with site excluded due not following the central randomization list, data of this site are highlighted in gray)

(A) Pooled analysis of IS revealed a reduction in IS with IPC (closed squares) when compared to plain I/R (open squares). **(B)** Site-specific analysis of IS revealed a reduction in IS with IPC in two sites, but no reduction in IS with IPC in one site. **(C)** Pooled analysis of AAR were comparable between I/R and IPC+I/R. **(D)** Site-specific analysis of AAR revealed a larger AAR in two sites. **(E)** Pooled analysis of myocardial edema revealed that IPC reduced myocardial edema when compared to plain I/R. **(F)** Site-specific analysis of myocardial edema were comparable between the 3 sites. **(G)** Pooled analysis of LVEF was comparable between I/R and IPC+IR. **(H)** Site-specific analysis of LVEF was comparable between 2 sites. Data are presented as minimum and maximum (whiskers), interquartile range from 25 to 75% (box), mean (square), median (line), and outlier (x) in a box plot and as intra-individual single data points. These data were not statistically analyzed, as data from an excluded center are presented.

AAR: area-at-risk; CMR: cardiac magnetic resonance imaging; IPC: ischemic preconditioning; I/R: ischemia/reperfusion; IS: infarct size; LV: left ventricle; LVEF: left ventricular ejection fraction

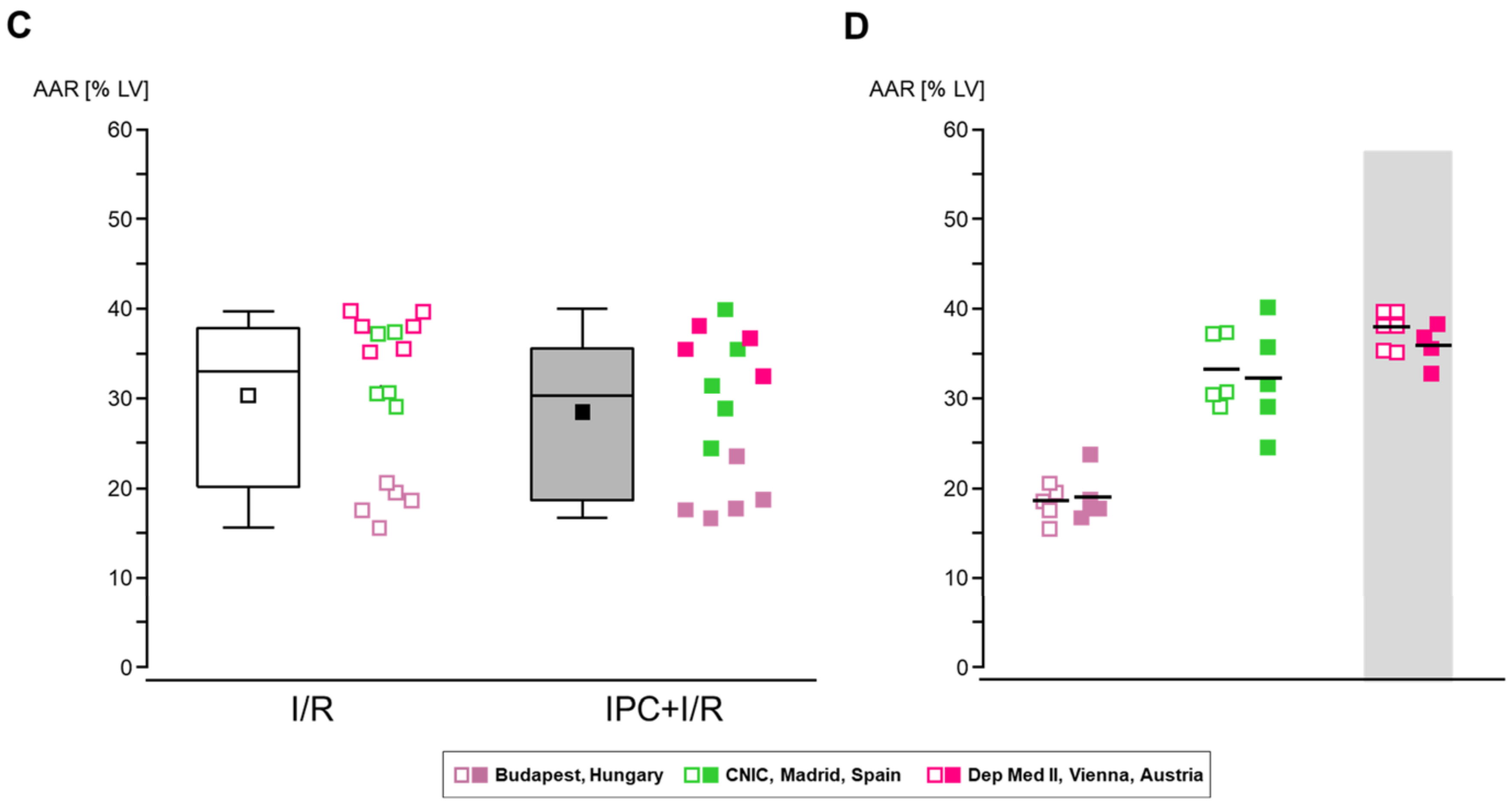

Supplemental Figure 5 - continued

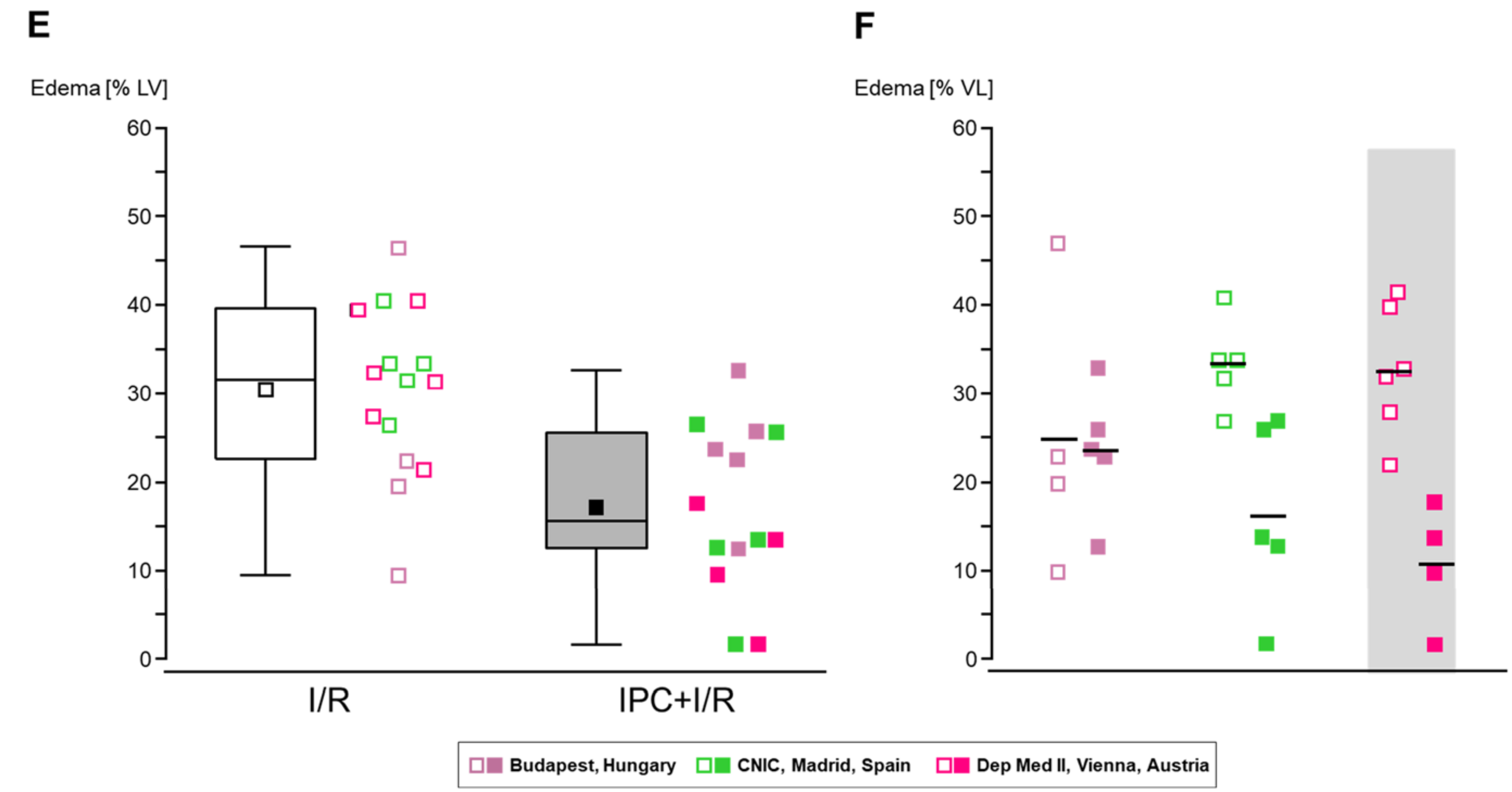

Supplemental Figure 5 - continued

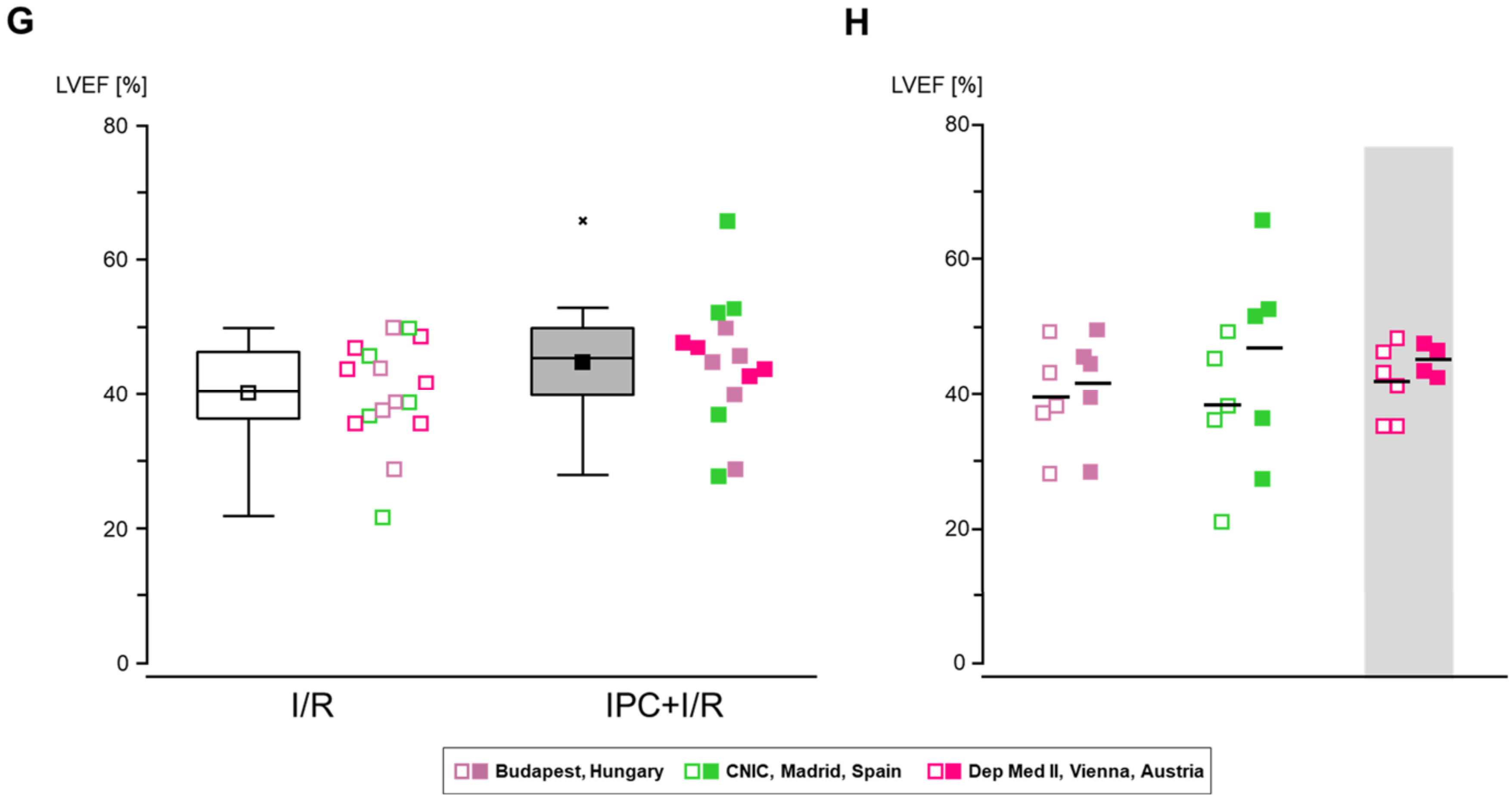

Supplemental Figure 5 - continued

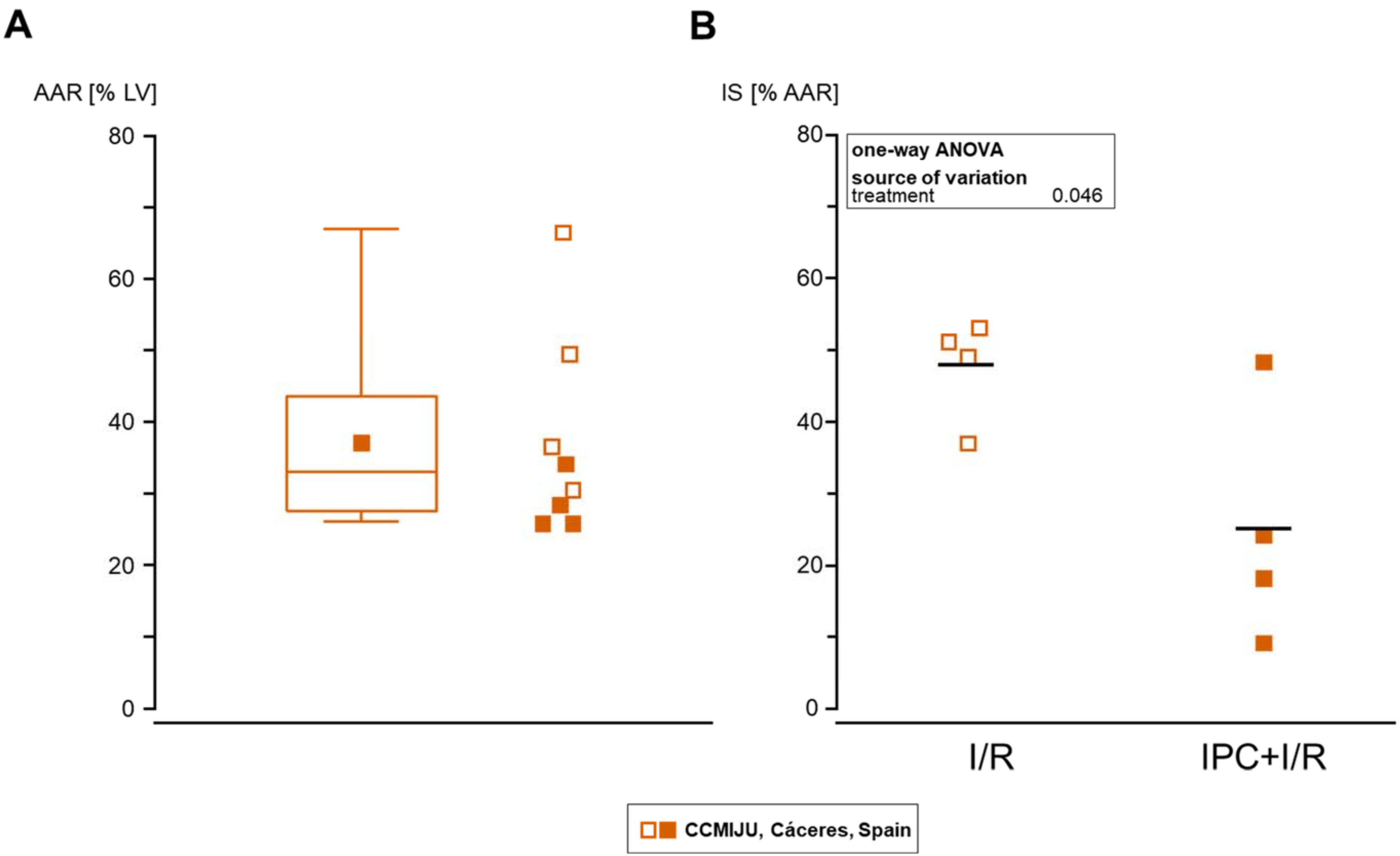

Supplemental Figure 6: Site-specific analysis of area-at-risk and infarct size (quantified via triphenyl-tetrazolium-chloride) for new studies performed by site originally excluded due to small IS on pre-defined criteria

(A) AAR are comparable between I/R (open squares) and IPC+I/R (closed squares). **(B)** IS was significantly reduced by IPC in comparison to plain I/R. Data are presented as minimum and maximum (whiskers), interquartile range from 25 to 75% (box), mean (square), and median (line) in a box plot and as intra-individual single data points.

AAR: area-at-risk; ANOVA: analysis of variance; IPC: ischemic preconditioning; I/R: ischemia/reperfusion; IS: infarct size; LV: left ventricle; TTC: triphenyl-tetrazolium-chloride
